# The microbiota metabolite indole inhibits *Salmonella* virulence: Involvement of the PhoPQ two-component system

**DOI:** 10.1371/journal.pone.0190613

**Published:** 2018-01-17

**Authors:** Nandita Kohli, Zeni Crisp, Rebekah Riordan, Michael Li, Robert C. Alaniz, Arul Jayaraman

**Affiliations:** 1 Department of Chemical Engineering, Texas A&M University, College Station, Texas, United States of America; 2 Department of Microbial Pathogenesis and Immunology, Texas A&M Health Science Center, College Station, Texas, United States of America; Robert Koch Institute, GERMANY

## Abstract

The microbial community present in the gastrointestinal tract is an important component of the host defense against pathogen infections. We previously demonstrated that indole, a microbial metabolite of tryptophan, reduces enterohemorrhagic *Escherichia coli* O157:H7 attachment to intestinal epithelial cells and biofilm formation, suggesting that indole may be an effector/attenuator of colonization for a number of enteric pathogens. Here, we report that indole attenuates *Salmonella* Typhimurium (*Salmonella*) virulence and invasion as well as increases resistance to colonization in host cells. Indole-exposed *Salmonella* colonized mice less effectively compared to solvent-treated controls, as evident by competitive index values less than 1 in multiple organs. Indole-exposed *Salmonella* demonstrated 160-fold less invasion of HeLa epithelial cells and 2-fold less invasion of J774A.1 macrophages compared to solvent-treated controls. However, indole did not affect *Salmonella* intracellular survival in J774A.1 macrophages suggesting that indole primarily affects *Salmonella* invasion. The decrease in invasion was corroborated by a decrease in expression of multiple *Salmonella* Pathogenicity Island-1 (SPI-1) genes. We also identified that the effect of indole was mediated by both PhoPQ-dependent and independent mechanisms. Indole also synergistically enhanced the inhibitory effect of a short chain fatty acid cocktail on SPI-1 gene expression. Lastly, indole-treated HeLa cells were 70% more resistant to *Salmonella* invasion suggesting that indole also increases resistance of epithelial cells to colonization. Our results demonstrate that indole is an important microbiota metabolite that has direct anti-infective effects on *Salmonella* and host cells, revealing novel mechanisms of pathogen colonization resistance.

## Introduction

The intestinal microbiota (the dynamic community of ~10^14^ microorganisms present in the human gastrointestinal (GI) tract) is an important mediator of several aspects of health, including promoting defense against pathogen colonization [1, 2]. The protective effect of the microbiota against pathogenic infections is termed as colonization resistance [3]. Several factors contribute to this phenomenon including competition between the indigenous microorganisms and the pathogen for nutrients [4, 5] and adhesion sites [6, 7], production of bacteriocins [8–10] and metabolites such as short chain fatty acids (SCFAs) [11–13] by the microbiota, and modulation of host defense mechanisms [1, 14]. It is well documented that alterations in the abundance and composition of the microbiota [15, 16] leads to an increased susceptibility to pathogen colonization [14].

Non-typhoidal *Salmonella* is among the top five causative pathogens of foodborne illness in the United States (Centers for Disease Control and Prevention, 2011 estimates). It is also the primary cause of hospitalizations and deaths, resulting from foodborne illnesses. *Salmonella* infection involves activation of two distinct Type III Secretion Systems (TTSS), essential for bacterial invasion and intracellular survival. These TTSSs are virulence factors encoded by *Salmonella* pathogenicity island 1 (SPI-1) and SPI-2, respectively, and are required for *Salmonella* infections [17, 18].

Pathogen virulence factors are known to be modulated by several microbiota-derived compounds. Of these, SCFAs are a well-studied class with an established role in the modulation of enteric infections by *Salmonella*, *Listeria*, *Campylobacter*, *Shigella*, and *E*. *coli* [19]. While propionate [13] and butyrate [11] decrease *Salmonella* virulence, formate [20] and acetate [21] have been shown to increase *Salmonella* virulence and infection. Previous work has shown that metabolites derived from tryptophan such as indole [22] are another class of molecules that inhibit colonization of pathogens like enterohemorrhagic *E*. *coli* (EHEC) and *Candida albicans* [23, 24]. On the other hand, indole has been shown to improve the survival of *E*. *coli* and *Salmonella* under antibiotic stress [25]; thus, pathogens that do not produce indole (such as *Salmonella*) can potentially benefit from indole-mediated signaling and lead to increased antibiotic resistance primarily through the OxyR regulon [26].

The molecular basis for the effects of indole on pathogenic bacteria is not fully understood. Nikaido et al [27] reported that indole induced expression of multidrug efflux pumps in *Salmonella*. Using a genome-wide analysis, they determined that indole exposure leads to a decrease in the expression of SPI-1 genes, reduction in flagellar motility and *in vitro* invasion, along with an increase in the expression of genes involved in efflux-mediated multidrug resistance [28]. They demonstrated that while the indole-mediated up-regulation of the AcrAB-TolC multidrug efflux system was RamA/RamR dependent, the down-regulation of virulence genes was not. Therefore, the mechanism(s) involved in mediating the effects of indole on *Salmonella* virulence is not clear.

In this study, we investigated the effect of indole exposure on *Salmonella* virulence and infection. A competitive index assay was used to compare the fitness of indole-treated and non-treated *Salmonella* in infecting mice. In addition, the effect of indole on other *Salmonella* functions important for infection such as motility, invasion, intracellular survival, and SPI-1 gene expression was also investigated. We also investigated the mechanism by which indole mediated down-regulation of *Salmonella* virulence and the combinatorial effect of indole on SPI-1 gene expression in the presence of SCFAs. Since we previously reported that indole attenuates host cell inflammation and increases intestinal epithelial cell barrier integrity [29], we further investigated the susceptibility or resistance of indole-conditioned epithelial cells, to *Salmonella* invasion. Our results suggest that tryptophan-derived microbiota metabolites could be important mediators of colonization resistance to *Salmonella* infection in the GI tract.

## Results

### Indole exposure decreases *Salmonella* invasion *in vivo*

**Fig 1:**
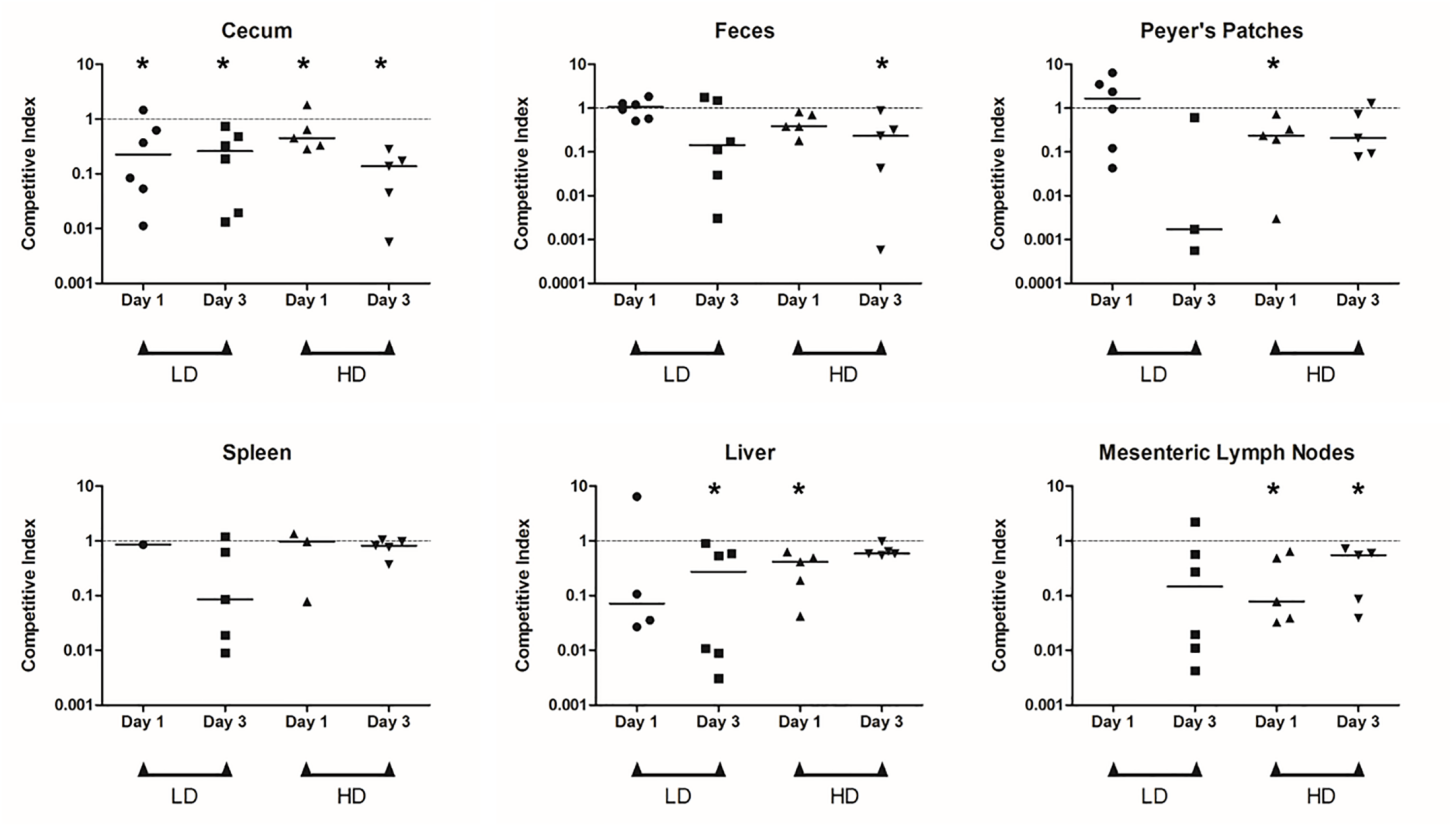
*In vivo* competition assays in C57BL/6 mice with indole treated *Salmonella*. Competitive index (CI) values for the indole treated *Salmonella* versus the control in different organs harvested from infected mice (n = 5) at days 1 and 3 post inoculation. Two inoculum doses were tested- low dose (LD; ~5 × 10^7^ cfu) and high dose (HD; ~5 × 10^8^ cfu) and several organs—cecum, Peyer’s patches, spleen, liver and mesenteric lymph nodes—were harvested. Feces were collected prior to euthanization. The organs were homogenized and serial dilutions plated to obtain cfu counts that were used to calculate the CI values. Each symbol (circle, square, upright triangle and downward triangle) on the plot represents a mouse from the respective group (LD day 1, LD day 3, HD day 1 and HD day 3, respectively). Lack of symbol indicates that no colonies were observed with that sample. For organs where indole treated *Salmonella* were absent but solvent treated *Salmonella* were present, CI was calculated assuming a cfu of 1 for the indole treated *Salmonella*. The horizontal bar represents the median of the observed CI values. * denotes significantly lower (*p* < 0.05) recovery of indole-treated *Salmonella* relative to solvent-treated *Salmonella*, as represented by the plotted CI values, using the Wilcoxon matched pair test.

A competitive index (CI) assay was used to determine the effect of indole on the ability of *Salmonella* to invade the murine GI tract. Fig 1 shows the CI of indole-treated *Salmonella* on day 1 and day 3 after infection for a low dose (LD) and high dose (HD) *Salmonella* inoculum. For the LD group, no significant difference between the counts of indole- and solvent-treated bacteria was observed in the Peyer’s patches (PPs) and feces on days 1 and 3 (S1A and S1B Fig). However, the number of indole-treated bacteria recovered in the cecum was significantly lower (*p* < 0.05) than the control on days 1 and 3 (Fig 1, S1A and S1B Fig). Indole-treated *Salmonella* was not detectable in the spleen and liver (Fig 1 and S1A Fig). On day 3, solvent-treated *Salmonella* were recovered from the spleen and liver of all mice; however, indole-treated *Salmonella* were recovered from livers and spleens of ~50% of the mice (S1B Fig). Both indole- and solvent-treated *Salmonella* were not recovered from mesenteric lymph nodes (MLN) on day 1. However by day 3, solvent-treated *Salmonella* were present in MLNs of all mice but indole-treated *Salmonella* were present in only 50% of the mice (Fig 1 and S1B Fig).

For the HD group, the number of indole-treated bacteria, recovered from the cecum was significantly lower (*p* < 0.05) than the number of solvent-treated bacterial numbers on both day 1 and day 3 post inoculation (Fig 1, S1C and S1D Fig). The counts of indole-treated bacteria were significantly lower (*p* < 0.05) in the PPs on day 1 and feces on day 3 (S1C and S1D Fig). No difference in the counts of indole- and solvent-treated *Salmonella* was observed in the spleen on days 1 and 3. The liver had significantly lower (*p* < 0.05) numbers of indole-treated bacteria compared to solvent-treated *Salmonella* on day 1, while the difference was less significant (*p* < 0.10) on day 3 (Fig 1 and S1C and S1D Fig). In the MLNs, significantly lower (*p* < 0.05) number of indole-treated *Salmonella* was detected compared to the solvent-treated *Salmonella* on day 1 and day 3.

### Indole decreases *Salmonella* motility

**Fig 2:**
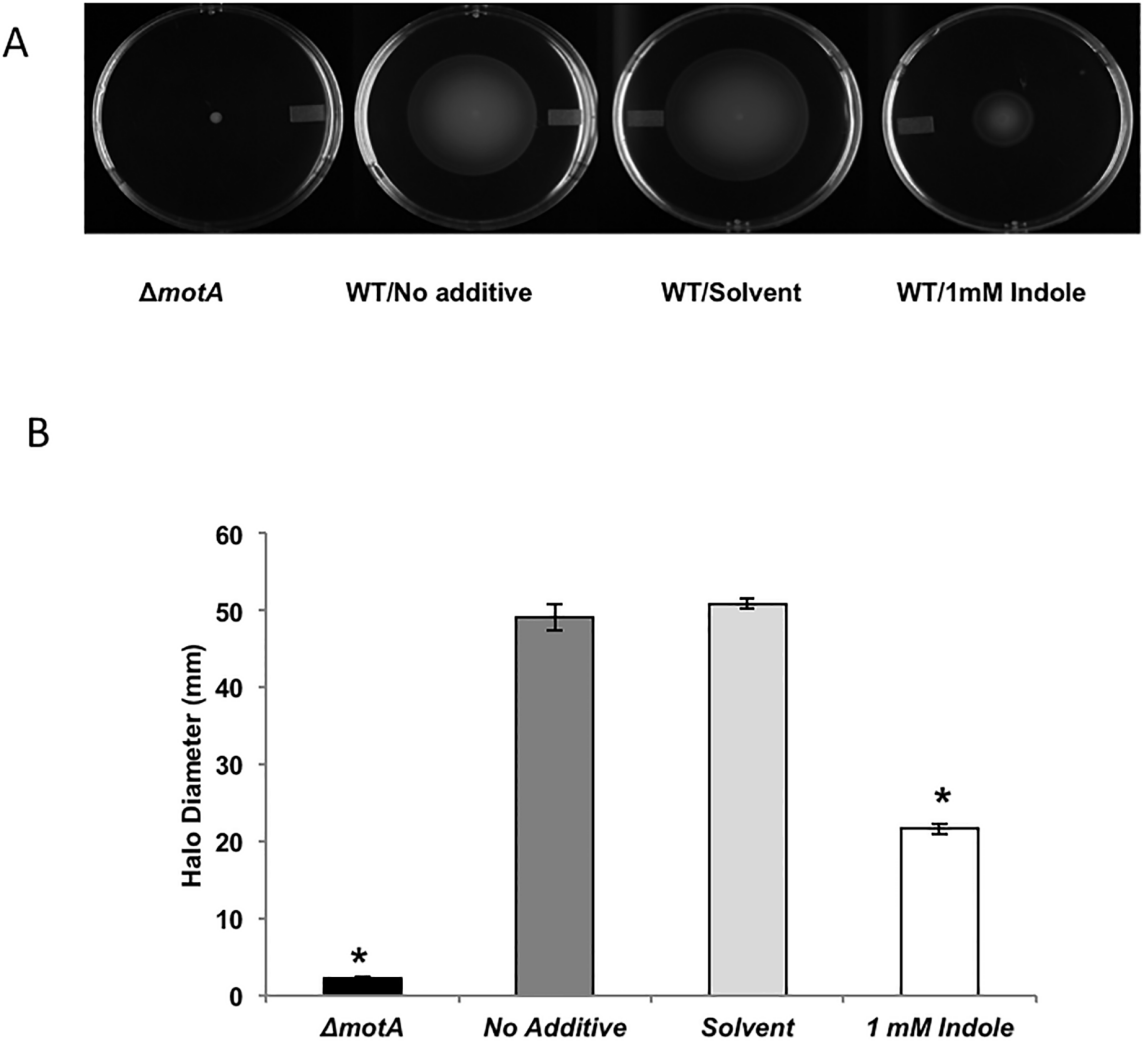
Effect of indole on *Salmonella* swimming motility at 37°C. (A) Representative photographs of the swimming motility agar plates spotted with WT *Salmonella*. (B) Measured halo diameters for the different test conditions. Diameters were measured using Vernier calipers, 8 hours post spotting. Δ*motA* was spotted on swimming motility agar plates as a negative control for motility. * denotes statistical significance relative to the solvent control at *p* < 0.05 using the Student’s *t*-test. Column bars depict mean (n = 4) and error bars represent standard deviation (SD).

Since motility is a virulence factor for enteric pathogens [30], we determined the effect of indole on *Salmonella* motility *in vitro* by measuring the halo diameter in the presence or absence of indole as a measure of motility. Exposure to indole at 37°C reduced *Salmonella* motility by ~ 60% as compared to solvent-treated controls (Fig 2). A similar inhibition in motility was observed when *Salmonella* were exposed to 1 mM indole at 30°C (~ 40% decrease in motility as compared to controls; see S2A Fig).

### Indole decreases *Salmonella* invasion but not its intracellular survival

**Fig 3:**
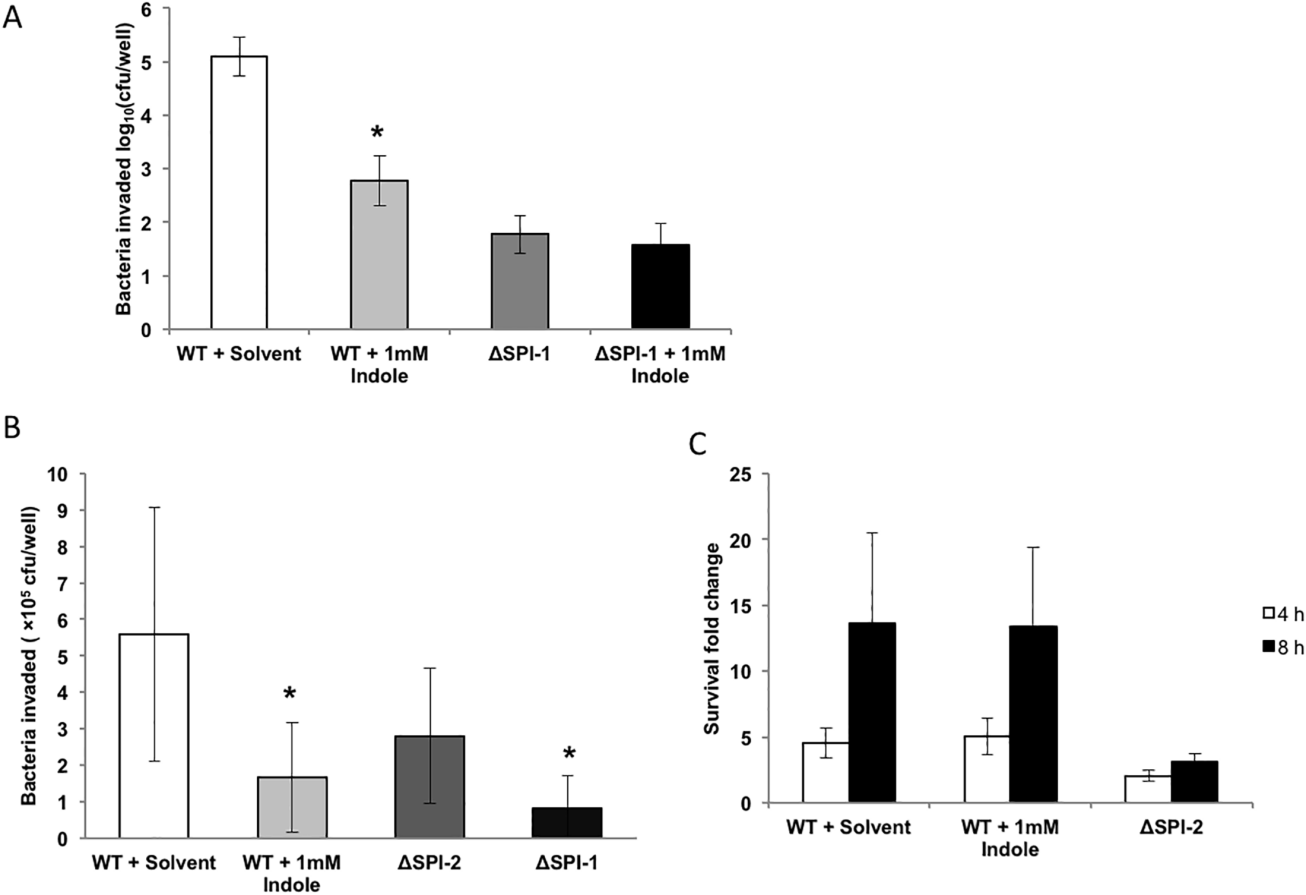
Invasion of epithelial cells and invasion and intracellular survival within macrophages with indole-treated *Salmonella*. Invasion in HeLa epithelial cell line (A) with *Salmonella* treated with or without 1mM indole. Invasion (B) and intracellular survival (C) in J774A.1 cells. Infection with the ΔSPI-1 and ΔSPI-2 strains were used as controls. A MOI of 50:1 was used for HeLa cells and a MOI of 10:1 was used for J774A.1 macrophages. Data shown are intracellular bacteria recovered and fold changes in survival (at 4 and 8 h post invasion) relative to the invasion. * denotes statistical significance relative to the solvent control at *p* < 0.05 using the Student’s *t*-test. Column bars depict mean (n = 3) and error bars represent standard deviation (SD).

We investigated the effect of indole on invasion of epithelial cells by *Salmonella*. A 160–fold decrease in invasion of the HeLa epithelial cell line was observed when *Salmonella* was treated with 1 mM indole prior to *in vitro* infection (Fig 3A). No change in invasion was observed with a SPI-1 mutant (ΔSPI-1) upon indole treatment. Since *Salmonella* invades and replicates inside macrophages after breaching the epithelial cell layer, we also investigated the effect of indole exposure on invasion and intracellular survival of macrophages. Fig 3B shows that *Salmonella* exposed to 1mM indole invaded J774A.1 murine macrophages approximately 2-fold less than the untreated controls. Fig 3C shows that indole exposure did not significantly alter intracellular survival in J774A.1 macrophages up to 8 h.

### Indole decreases *Salmonella* virulence gene expression

**Fig 4:**
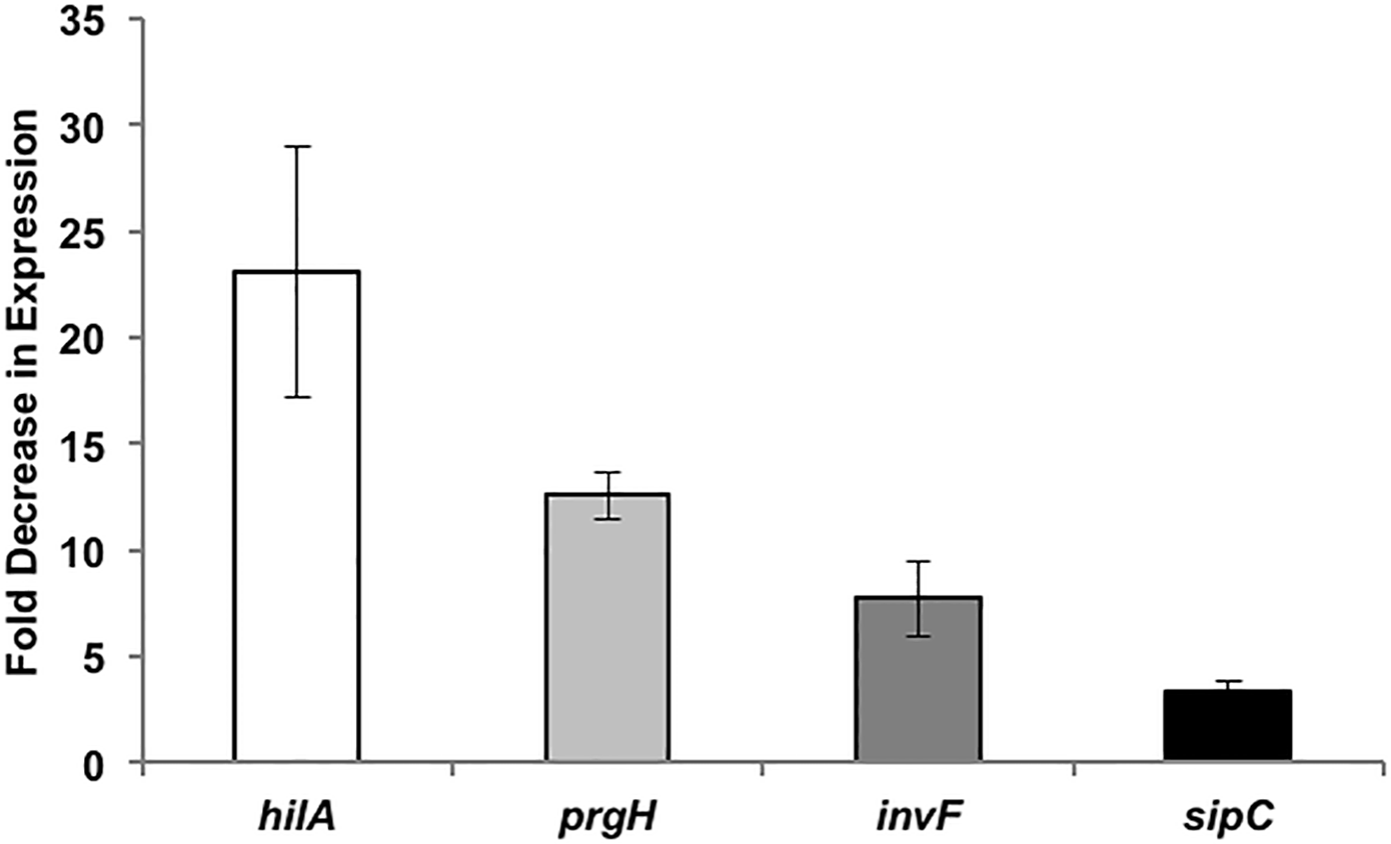
SPI-1 virulence gene expression change in WT *Salmonella* upon treatment with 1 mM indole. SPI-1 reporter strains for *hilA*, *prgH*, *invF* and *sipC* were treated overnight with and without 1 mM indole and the ß-gal activity was measured in exponential phase cultures after dilution. Data shown are the mean fold decrease in expression (n = 3) with indole-treatment relative to the solvent-treated control at a significance level of *p* < 0.05 using the Student’s *t*-test. Error bars represent SD.

A ß-gal reporter assay was used to determine whether the decrease in invasiveness of *Salmonella* was mirrored by changes in the expression of genes in the *Salmonella* pathogenicity island-1 (SPI-1). Fig 4 shows that the expression of *hilA*, *sipC*, *invF*, and *prgH* were all down-regulated to different degrees upon exposure to 1 mM indole. The expression of *hilA* was decreased significantly by 23-fold upon exposure to indole, whereas the expression of *prgH*, *invF*, and *sipC* decreased by 12-, 8- and 3-fold, respectively. Therefore, the reduced expression of genes involved in the invasion process was consistent with the decrease in invasion of epithelial cells by *Salmonella* upon indole treatment.

### Role of *phoPQ* in the indole-mediated decrease in virulence

**Fig 5:**
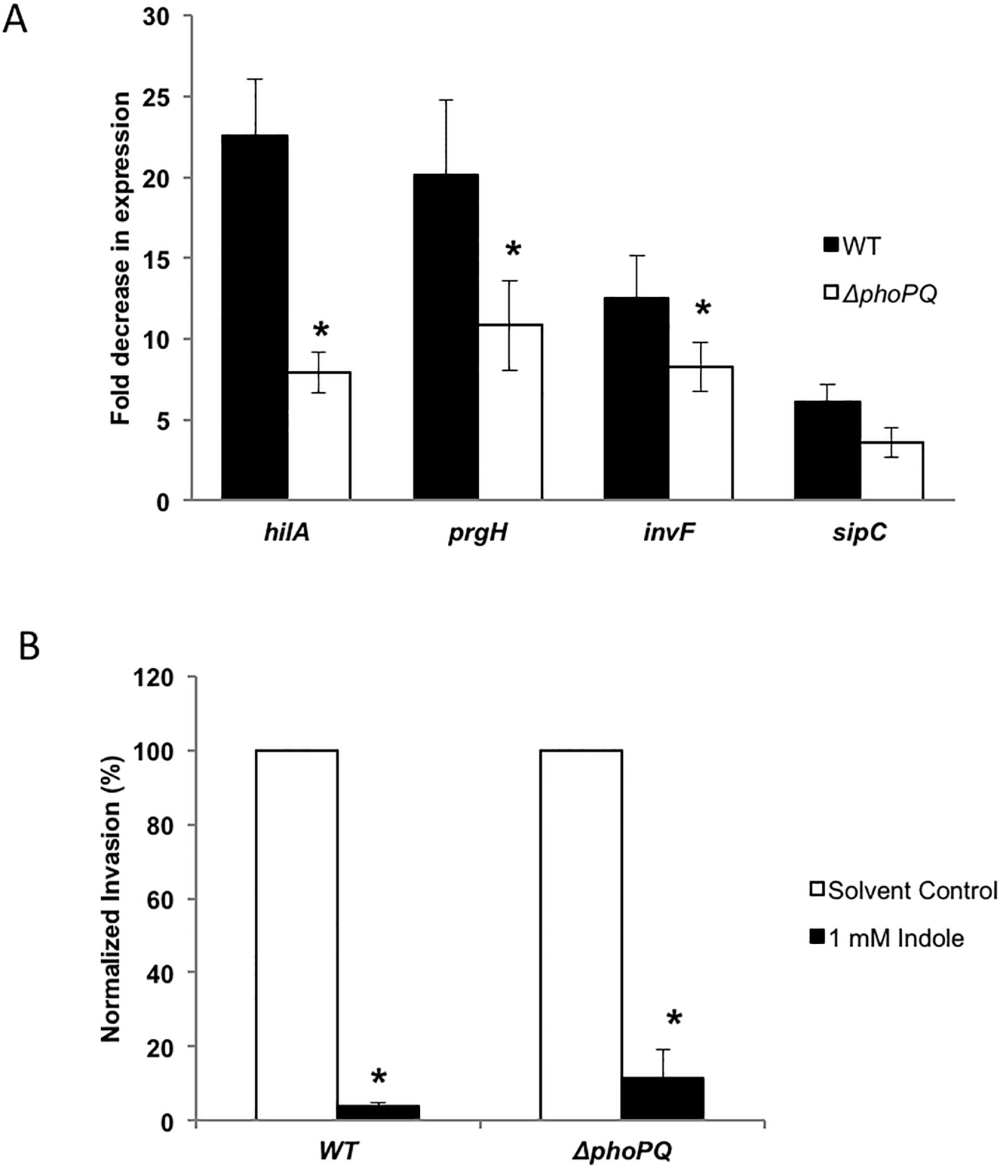
Role of *phoPQ* in indole mediated down-regulation of virulence. (A) SPI-1 virulence gene expression using ß-gal assay. The Δ*phoPQ* mutation was generated in the four SPI-1 reporter strains for *hilA*, *prgH*, *invF* and *sipC*. The WT and the Δ*phoPQ* reporter strains were treated overnight with and without 1 mM indole and the ß-gal activity was measured in exponential phase cultures after dilution. Data shown are the fold decrease in expression with indole-treatment relative to the solvent-treated control. * denotes statistical significance relative to the WT strain at *p* < 0.05 using the Student’s *t*-test. (B) Invasion in HeLa epithelial cell line with *Salmonella* WT and Δ*phoPQ* strain treated with or without 1mM indole. A MOI of 100:1 was used and the data shown is the indole-treated *Salmonella* invasion normalized to the control of the respective strain. * denotes statistical significance with respect to the solvent control at *p* < 0.05 using the Student’s *t*-test. Column bars depict mean (n = 3) and error bars represent standard deviation (SD).

*Salmonella* with a constitutively expressed *phoP* (part of the *phoPQ* two-component signaling system) is known to reduce the expression of *prg* genes [31]. We investigated whether the effect of indole was mediated through the *phoPQ* two-component system. Exposure to 1 mM indole decreased the expression of the four SPI-1 genes tested (*hilA*, *prgH*, *invF* and *sipC*) by 8-, 11-, 8- and 4-fold, respectively, in the Δ*phoPQ* mutant; however, the magnitude of attenuation was ~2-fold less than that observed in wild type cells i.e. 23-, 20-, 13- and 6-fold, respectively, for *hilA*, *prgH*, *invF* and *sipC* (see Fig 5A). This suggests that *phoPQ* decreases SPI-I gene expression and *Salmonella* virulence using PhoPQ-dependent and independent mechanisms. Epithelial cell invasion assays with the Δ*phoPQ* mutant were consistent with this observation as the decrease in invasion with the Δ*phoPQ* mutant upon indole treatment was ~ 9-fold, which was ~ 3-fold less than that observed for the WT strain (~ 26-fold) (see Fig 5B).

### Indole synergizes with SCFAs

**Fig 6:**
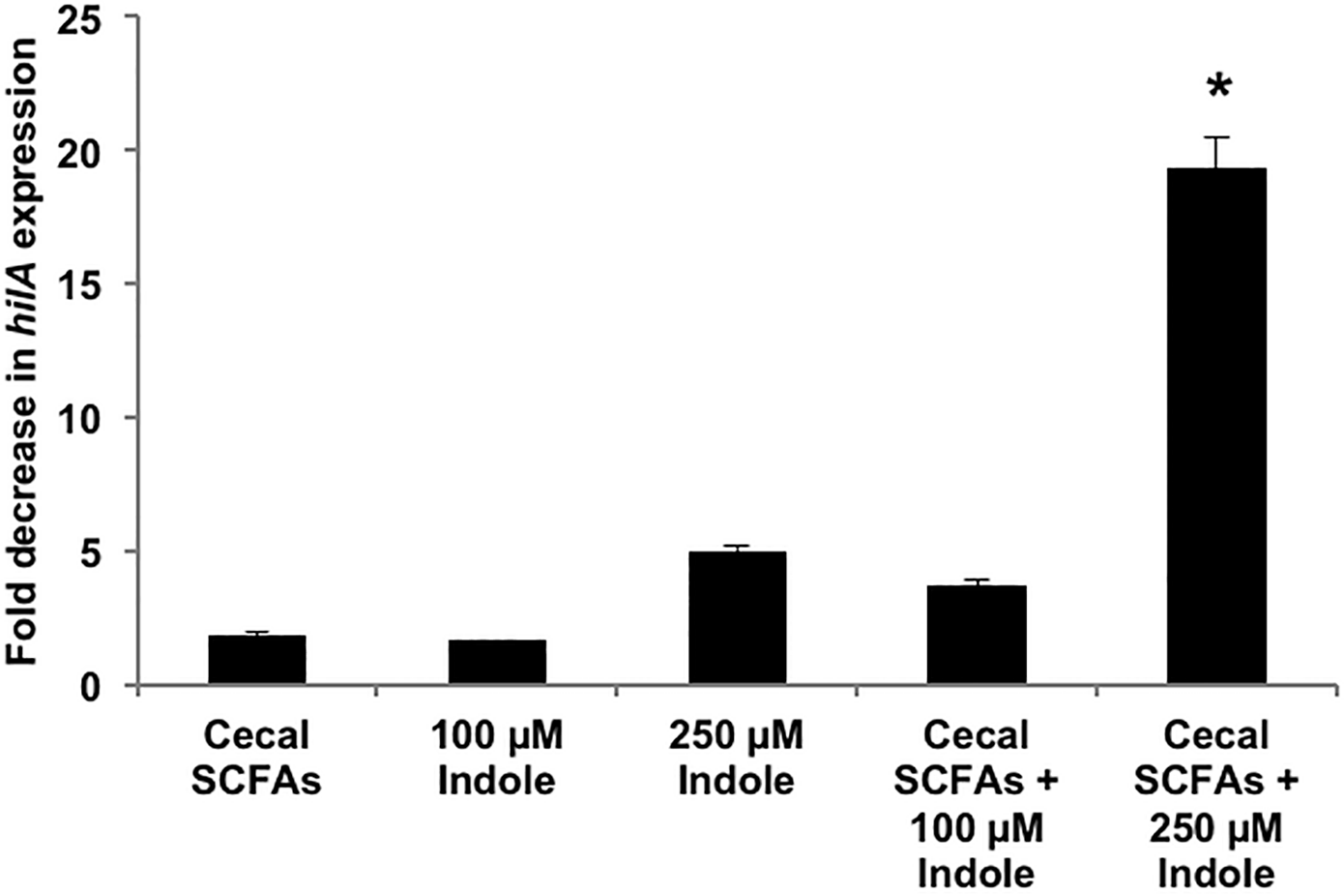
Effect of indole in combination with cecal SCFAs on *hilA* expression. SPI-1 reporter strain for *hilA* was treated overnight with and without indole (100 μM and 250 μM) in the presence of 200 mM cecal SCFAs or 200 mM NaCl, and the ß-gal activity was measured in exponential phase cultures after dilution. Data shown are the mean fold decrease (n = 3) in expression of *hilA* with treatment relative to the control: *hilA* expression in presence of 200 mM NaCl. * denotes statistical significance relative to cecal SCFAs alone at *p* < 0.05 using the Student’s *t*-test. Error bars represent SD.

Given the likely interactions among GI tract metabolites to mediate colonization resistance, we hypothesized indole’s effect on *Salmonella* virulence may be augmented when present along with other GI tract microbiota metabolites. We specifically focused on short chain fatty acids (SCFAs) as they are abundant in the GI tract [21, 32–34] and are important modulators of pathogen virulence [19]. Therefore, we investigated the combined effect of indole (100 μM and 250 μM) and SCFAs (110 mM acetate, 70 mM propionate and 20 mM butyrate for a total concentration of 200 mM) on *hilA* expression. The average fold decrease in *hilA* expression upon treatment with cecal SCFAs alone was 1.8-fold and the decrease in *hilA* expression with 100 μM and 250 μM indole alone was 1.6- and 5.0-fold, respectively (Fig 6). However, when 100 μM or 250 μM indole was present with cecal SCFAs, the observed average decrease in *hilA* expression was 3.7-fold and 19.3-fold, respectively. These observations suggest that indole enhances the down-regulatory effect of cecal SCFAs on *hilA* expression in an additive (100 μM indole) or syngeristic (250 μM indole) manner.

### Indole increases epithelial cells resistance to *Salmonella* invasion

**Fig 7:**
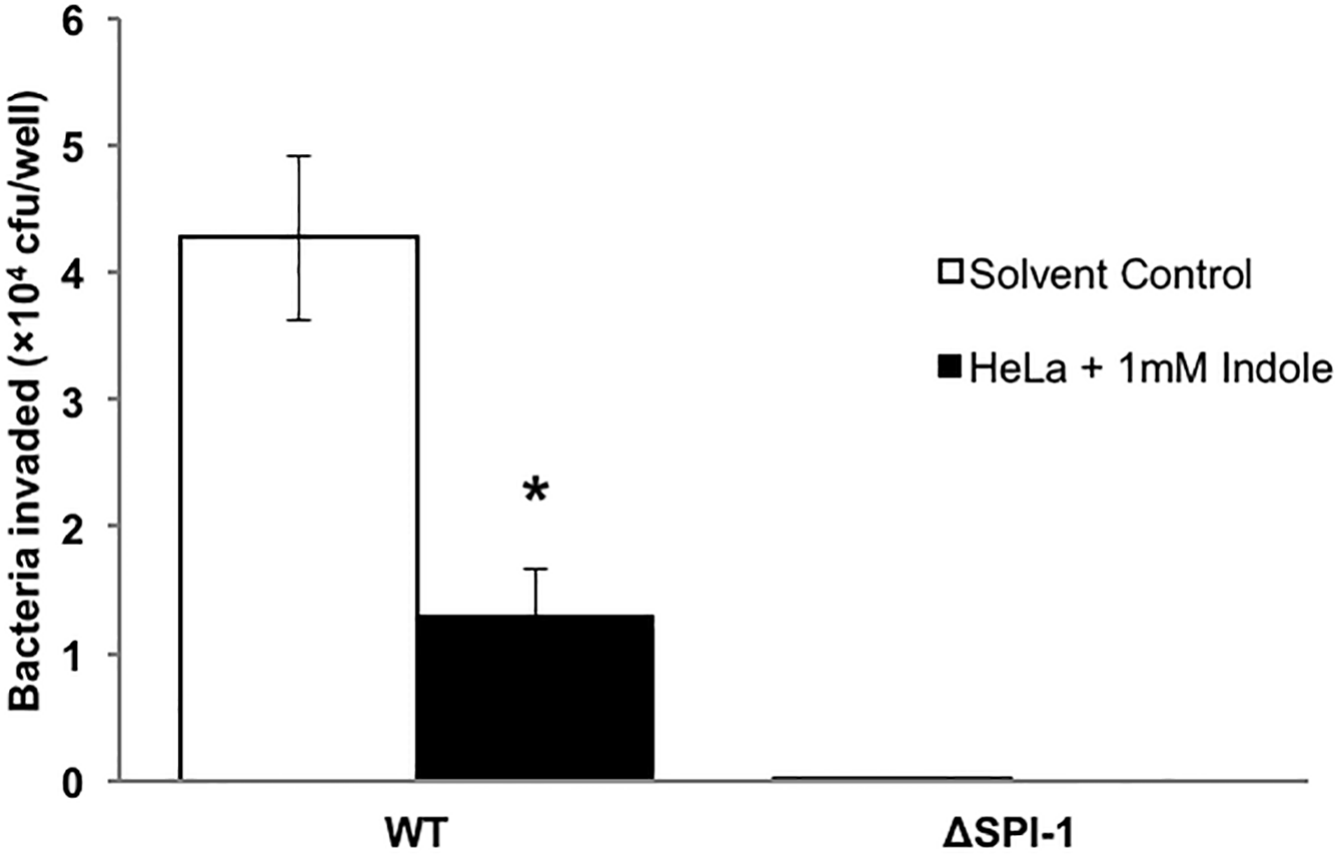
Effect of indole on colonization resistance in HeLa epithelial cells. HeLa cells were seeded in a 24 well plate and conditioned with 1 mM indole for 24 h prior to infection. A MOI of 10:1 was used for infection. Data shown are intracellular bacteria recovered from infected HeLa monolayers with indole treatment or control (solvent treatment). * denotes statistical significance relative to the solvent control at *p* < 0.05 using the Student’s *t*-test. Column bars depict mean (n = 3) and error bars represent SD.

To determine whether indole also impacted the ability of host cells to resist *Salmonella* invasion, we exposed HeLa epithelial cells to indole prior to infection with *Salmonella* (not exposed to indole) and determined the extent of *Salmonella* invasion. Fig 7 shows that a statistically-significant 70% decrease in invasion was observed when indole-conditioned epithelial cells were infected with wild type *Salmonella*, compared to untreated HeLa cells. This suggests that indole increases resistance of host cells to *Salmonella* invasion in addition to attenuating *Salmonella* virulence.

## Discussion

The link between prevention of pathogen colonization and the GI tract microbiota has been long established [3], and a number of contributing factors such as nutrient competition [5], steric hindrance [35], production of bacteriocins [8–10] and specific metabolites such as SCFAs [11–13] have been reported to play a role in this phenomenon [1, 2]. However, besides SCFAs, few other specific classes of molecules have been identified that impact pathogen colonization. Here, we report that indole, an abundant tryptophan-derived microbiota metabolite, attenuates *Salmonella* infectivity *in vivo* and virulence *in vitro*, as well as increases resistance of host cells to *Salmonella* invasion *in vitro*.

Indole is produced from tryptophan by the enzyme trytophanase (TnaA) [36] that is present in *E*. *coli* and several other microorganisms present in the GI tract belonging to the phyla- *Bacteroidetes*, *Firmicutes*, *Proteobacteria* and *Actinobacteria*- [25]. Indole is an abundant microbiota metabolite in the GI tract luminal microenvironment where pathogen colonization is initiated. Indole concentrations of ~40 nmol/g tissue in murine cecum were reported by Whitt et al. using an enzymatic assay [37]. Recently, we used mass spectrometry to determine that indole is present at 10–40 nmol/g sample wet weight in murine cecum. Based on unpublished data from our lab that the extraction efficiency of indole from cecal contents is ~15% and assuming that cecal contents have a density similar to that of water, the effective concentration of indole in cecal contents is ~100–300 μM. Another recent study determined fecal indole levels in 53 healthy adults to vary from 0.3 mM to 6.64 mM with a mean of 2.59 mM [38] (i.e., comparable to concentrations at which a response was observed in this study).

The reduced colonization *in vivo* by indole-treated *Salmonella* in mice is apparent from the statistically significant difference in the number of indole-treated and non-treated *Salmonella* detected in the cecum for both the LD and HD groups post infection (S1 Fig). Although in our study, *Salmonella* were exposed to indole prior to infection, both the strains (indole-treated and solvent-treated) encountered the same environment *in vivo* with the only difference being the preceding indole exposure. Our observations suggest that comparatively fewer indole-treated *Salmonella* invaded the intestinal epithelium and colonized the cecum, leading us to conclude that indole exposure lowers *Salmonella*’s capability to colonize *in vivo*. Since the cecum is reportedly a reservoir for *Salmonella* intestinal persistence and fecal shedding in mice [39, 40], it is interesting to observe the lower competitiveness of indole-treated *Salmonella* to colonize the cecum with CI < 1 (Fig 1). Gnotobiotic studies co-colonizing mice with *Salmonella* and an indole over-producer strain or a deficient mutant would help to further elucidate indole’s role *in vivo*.

The marked decrease in *Salmonella* motility, invasion of epithelial cells and macrophages, and decrease in virulence gene expression upon exposure to indole is similar to our previous report on indole’s effect on EHEC motility, biofilm formation, and its colonization of epithelial cells [23]. However, to our knowledge, this is the first *in vivo* study demonstrating that indole’s effect on pathogen virulence translates to reduced infectivity in mice. A striking aspect of our results is the concordance between observations at multiple levels or stages of *Salmonella* infection. Another interesting observation is the temporal coordination in the effect of indole on SPI-I gene expression. The *hilA* gene is the master regulator of the SPI-I regulon [41] and an indole-mediated decrease in expression of *hilA* was observed first, when a time-course study was conducted, followed by decrease in expression of *prgH*, *invF* and *sipC*. HilA is a transcriptional regulator which activates the expression of structural type III secretion genes such as *prgH* and the transcription factor *invF* [41]. *SipC*, on the other hand, is a secreted effector (translocase) that is activated by *invF*. Thus, the reduced invasion *in vitro* and infectivity *in vivo* are likely the result of coordinated decrease in SPI-1 gene cluster expression.

While indole markedly attenuated invasion and the expression of SPI-I genes, it did not significantly affect intracellular survival of *Salmonella* in macrophages. This lack of effect on intracellular survival was also mirrored by lack of a significant change in the expression of SPI-II genes (*ssrB* and *ssaR*; S1 Table). The lack of effect on intracellular survival suggests that indole primarily modulates extracellular infection. The intracellular phase of *Salmonella*’s infection cycle allows *Salmonella* within macrophages to escape from Peyer’s patches to the lymph nodes and spread to the liver and spleen resulting in systemic disease. Distal ileum (in proximity to the cecum), with Peyer’s patches rich in lymphoid cells, is considered to be the primary enteric site for *Salmonella* infection causing systemic disease [42]. The CI < 1 observed for the systemic organs such as liver, spleen and mesenteric lymph nodes (Fig 1), is likely a result of the initial lower invasion and colonization by indole-treated *Salmonella*, and is consistent with our *in vitro* results showing that indole did not modulate intracellular survival.

The mechanism(s) underlying indole’s effects on pathogen virulence are poorly understood. Few transcriptional regulators and two-component systems have been reported to be involved in indole signaling. Kanamaru et al., [43] showed that the expression of virulence factors in EHEC is controlled by *sdiA* and that indole acts through *sdiA* [44]. However, our *in vitro* data with motility and invasion suggest that SdiA is not involved in mediating indole’s effects in *Salmonella*. The decrease in motility of a Δs*diA* mutant upon indole treatment was comparable to the wild-type strain at 37°C and 30°C (S2B and S2C Fig). These results indicate that indole’s effect on *Salmonella* motility is not mediated through *sdiA*. The decrease in invasion of HeLa epithelial cells and J774A.1 macrophages (and the lack of effect on intra-cellular survival) with indole-treated Δs*diA* mutant was also similar to that observed by the WT strain (S3 Fig), which further confirmed that *sdiA* is not involved in indole-mediated effects on *Salmonella*.

Several bacterial two-component systems sense environmental signals and one such regulatory system, *phoPQ*, has been reported to down regulate SPI-1 gene expression [31, 45, 46]. Our data indicate that the *phoPQ* two-component system is at least partially involved in mediating the effects of indole in *Salmonella*, as the change in expression of SPI-I genes upon indole exposure was neither unaltered nor completely abrogated in the Δ*phoPQ* mutant strains compared to the wild-type. Complementation of a Δ*phoQ* mutant with *phoQ* on a plasmid restored indole’s down-regulatory effect on *hilA* expression although not to the same level as in the WT strain (S4 Fig). These results also suggest that other pathways may be involved in indole mediated signaling that regulate virulence gene expression. Indole might interact with a second receptor and the partial effect observed in a *phoPQ* deletion mutant might be attributed to this secondary interaction. Another study on indole’s effect on *Salmonella* by Nikaido et al [28] found that while *ramA* is involved in indole signaling, the down-regulation of virulence gene expression with indole was independent of RamA/RamR. Therefore, while our data clearly shows a role for *phoPQ* in the down-regulation of *Salmonella* virulence by indole, further work needs to be done to fully elucidate the additional underlying mechanism(s).

Although we observed strong attenuation of *Salmonella* virulence and invasion with indole, it should be noted that several other metabolites can be derived by the microbiota from dietary tryptophan, and are present in the lumen of the GI tract such as indole-3-acetate, indole-3-pyruvate and tryptamine [22]. However, not all tested metabolites had the same effect on *Salmonella* as indole (S5 Fig). Indole-3-pyruvic acid decreased *hilA* expression by 3-fold whereas tryptamine and indole-3-acetic acid down-regulated *hilA* expression by 1.3- and 1.5-fold, respectively. Thus, there appears to be some variability in the anti-infective effect of microbiota-derived tryptophan metabolites. Further structure-function studies are required to identify feature(s) that are required to elicit the observed phenotype.

Apart from tryptophan metabolites, SCFAs constitute the other major class of microbiota metabolites abundant in the gut lumen. The total concentration of the SCFAs varies along the length of the GI tract- low (~20 mM) in the ileum and high (~140–200 mM) in the cecum and the colon [32–34]. The relative concentration of the individual components- acetate, propionate and butyrate- also varies in the ileal and colonic segments. Since SCFAs are known modulators of *Salmonella* virulence [11, 13, 20, 21], our data on the synergy between indole (at a concentration of 250 μM) and SCFAs in down-regulating *hilA* expression further underscores the importance of indole as a potent virulence-attenuating signal in the GI tract.

In addition to decreasing pathogen virulence phenotypes, we also observed that exposing epithelial cells to indole decreased *Salmonella* invasion. This suggests that indole (and presumably, other microbiota metabolites) could attenuate pathogen invasion and colonization by both inhibiting virulence directly in the pathogen and simultaneously increasing the resistance of host cells. This observation is also consistent with previous work from our laboratory showing that indole increased anti-inflammatory cytokine production and epithelial cell tight junction resistance in HCT-8 enterocytes [29]. In this regard, indole is similar to the SCFA butyrate in its scope of action. Butyrate is a major source of energy for colonocytes [47, 48] and inhibits bacterial pathogenesis through its effect on colonocytes as demonstrated by studies with *Campylobacter jejuni* [12]. Current work in our laboratory is focusing on elucidating the mechanism(s) underlying indole’s effect on host cells.

In summary, our observations demonstrate indole’s role in inhibiting *Salmonella* virulence and colonization. Taken together with our prior work showing that indole attenuates inflammatory gene expression in intestinal epithelial cells, our results suggest that microbiota metabolites such as indole could play an important role in determining the susceptibility of the host to pathogen infection in the GI tract. Since indole is also a chemorepellent for EHEC [23], it is intriguing to speculate that in addition to attenuating *Salmonella* virulence, indole also attenuates the recruitment and directed migration of *Salmonella* to its infection niche in the GI tract.

## Materials and methods

### Bacterial strains, cell lines, media and chemicals

*Salmonella enterica* serovar Typhimurium (ATCC 14028s) was grown and maintained in Luria-Bertani (LB) medium at 37°C supplemented with appropriate antibiotics where necessary. *Salmonella* SPI-1 reporter strains for *hilA*, *prgH*, *invF* and *sipC* [49] were a kind gift from Dr. Sara D. Lawhon. The ΔSPI-1, ΔSPI-2, Δ*motA* and Δ*sdiA* deletion mutants [50] and the isogenic Nalidixic acid resistant (Nal^R^) [51] strains were generous gifts from Dr. Helene Andrews-Polymenis. The Nal^R^ strain has been reported to be equally virulent as the ATCC14028 in murine models [51].

For all indole exposure experiments, cells were grown in LB overnight with or without indole, diluted to an O.D._600nm_ of ~0.05 and further grown for ~2 h in a shaker incubator (New Brunswick Scientific) at 37°C, 250 rpm to obtain an exponential phase culture (O.D._600nm_ of ~1.0), unless stated otherwise. 70% ethanol was used as the solvent control.

The murine macrophage cell line J774A.1 (ATCC), was maintained in the RPMI (Roswell Park Memorial Institute) 1640 medium with 10% fetal bovine serum, 1 mM sodium pyruvate, 10 mM HEPES, 2 g/L sodium bicarbonate, 0.05 mM 2-mercaptoethanol, 100 U/ml penicillin and 100 μg/ml streptomycin, at 37°C in 5% CO_2_. The HeLa cell line (ATCC) was maintained in DMEM (Dulbecco’s Modified Eagle Medium) supplemented with 10% bovine serum, 100 U/ml penicillin and 100 μg/ml streptomycin and 2 g/L sodium bicarbonate at 37°C in 5% CO_2_ during normal growth and culture.

### Generation of *Salmonella* deletion mutants

The Δ*phoPQ* and Δ*phoQ* mutations were generated in the *Salmonella* wild type and SPI-1 reporter strains using the Datsenko and Wanner method [52]. Briefly, gene deletion fragments encoding the kanamycin resistance gene flanked by upstream and downstream regions of gene to be deleted were generated using the designed primers and pKD13 plasmid as template (Table 1). The DNA fragments were purified and the desired fragment length product was digested with *DpnI* followed by purification. These were then electroporated into the wild-type *Salmonella* and SPI-1 reporter strains containing the pKD46 plasmid encoding recombinase. The recombinant deletion mutants were then selected using kanamycin and verified for the gene deletion using PCR.

**Table 1:** Primers used in this study.

Primer name	Sequence (5'—3')
Primers for generation of *phoPQ* and *phoQ* deletions	
*phoP*::Kan Forward	CATAATCAACGCTAGACTGTTCTTATTGTTAACACAAGGGAGAAGAGATGATTCCGGGGATCCGTCGACC
*phoQ*::Kan Reverse	GAGATGCGTGGAAGAACGCACAGAAATGTTTATTCCTCTTTCTGTGTGGGTGTAGGCTGGAGCTGCTTCG
*phoQ*::Kan Forward	GTCATTACCACCGTACGCGGACAAGGATATCTTTTTGAATTGCGCTAATGATTCCGGGGATCCGTCGACC
Primers for verification of *phoPQ* and *phoQ* deletions	
*phoP* Upstream Forward	ATTATATCGGTCGCGCTGTG
*phoQ* Downstream Reverse	AGAAAGTCGGGCCAGTTAAG
*phoP* Forward	GATGAAGACGGCCTTTCCTT
*phoQ* Reverse	GGCGATCCACAGTAAAGGAA
K1 Reverse [52]	CAGTCATAGCCGAATAGCCT
Primers for cloning *phoQ* in pCA24N plasmid	
N-terminal	GCCAATAAATTTGCTCGCCATTT
C-terminal	CCTTCCTCTTTCTGTGTGGGATG
Primers for SPI-2 gene expression	
*ssrB* Forward	GCGAGCGTCAGGTTCTTAAA
*ssrB* Reverse	CTCATTCTTCGGGCACAGTT
*ssaR* Forward	TTTCCTTAAACTGGCGGTGG
*ssaR* Reverse	ACTCAGACGTCCAGAAAGGA
*gyrB* Forward	CTGAACGCCTACATGGACAA
*gyrB* Reverse	CTGTTCTACCGCCGATTTCA

The *Salmonella phoQ* gene was cloned onto pCA24N plasmid using the strategy outlined in [53]. Briefly, the *Salmonella* Typhimurium *phoQ* gene was amplified with gene specific primers (Table 1) using NEB Phusion^®^ High-Fidelity DNA polymerase. The amplified gene fragments were ligated into the *Stu*I digested pCA24N using T4 DNA ligase. The ligated vector was transformed into chemically competent *E*.*coli* DH5-alpha cells. The plasmids were isolated from transformants and verified for presence of desired clone using *Sfi*I restriction and agarose gel electrophoresis.

### Motility assay

Motility assays were performed as described by Bansal et al [23]. Briefly, *Salmonella* was cultured in LB medium at 37°C or 30°C to exponential phase. Indole (1 mM) in 70% ethanol or the equivalent volume of solvent was added to motility agar plates (1% tryptone, 0.25% NaCl, and 0.3% agar), and the sizes of the motility halos were measured after 8 h. Four motility plates were used for each condition. A *motA* mutant was used as the negative control. Images were obtained using the Bio Rad VersaDoc imaging system model 3000.

### *In-vitro* invasion assay and intracellular survival assay

HeLa cells were cultured in a 24-well tissue culture plate at a cell density of ~5 × 10^5^ cells/well and infected with late log phase *Salmonella* cells at an MOI ~ 50:1 for 1 h to allow invasion. At the end of the incubation period, the media was replaced with medium containing gentamicin (100 μg/mL) and incubated for an additional hour to kill the *Salmonella* cells that did not invade. The HeLa cell monolayers were then washed twice with PBS and cells lysed with a 0.2% sterile solution of NP40 to release the invaded bacteria. The lysate was serially diluted and spread on LB agar plates to determine the number of invaded bacteria. The starting inoculum was also plated to obtain the initial count of bacterial cells used for infection. The percentage invasion was calculated as the ratio of bacterial cells invaded to cells inoculated.

For experiments testing the resistance of indole-conditioned HeLa cells to *Salmonella* infection, HeLa cells were treated with 1 mM indole for 24 h prior to infection. Cells were then washed twice to remove residual indole and fresh media without indole was added prior to infection at an MOI ~ 10:1. Indole does not alter viability of epithelial cells for the duration of exposure and at the concentration tested.

J774A.1 macrophage cells were also used for invasion and intracellular survival assay. Cells were plated in a 24 well plate at a density of ~5 × 10^5^ cells/well and treated with serum-free RPMI medium overnight to synchronize them in a quiescent state. Prior to infection, the serum-free medium was replaced with RPMI medium supplemented with 10% heat-inactivated serum. The protocol for the invasion assay was similar to that used for HeLa cells, except that a lower MOI ~10:1 was used since the macrophages are inherently phagocytic.

The intracellular survival of *Salmonella* at 4 h and 8 h post-invasion was determined by incubating the invaded J774A.1 cells in heat-inactivated serum RPMI media supplemented with 5μg/mL gentamicin at 37°C, 5% CO_2_. Intracellular bacterial counts were obtained by lysing J774A.1 cells and plating serial dilutions on LB agar plates. The extent of survival was calculated as the ratio of the surviving intracellular bacteria to the number of bacteria that invaded.

### *Salmonella* SPI-1 reporter assays

*Salmonella* SPI-1 reporter strains for *hilA*, *prgH*, *invF* and *sipC* with the ß-galactosidase (ß-gal) gene fused to each gene [49], were grown overnight in LB at 37°C and 250 rpm. Cells were diluted to an O.D._600_ of ~0.05 in LB with 1 mM indole and grown to exponential phase, unless stated otherwise. ß-gal activity measurements were made for the collected samples using a fluorogenic substrate (Resorufin ß-D-galactopyranoside, AnaSpec) using a microplate scanning spectrofluorometer (SpectraMax, Gemini EM, Molecular Devices) with excitation and emission wavelengths as 544 nm and 590 nm, respectively. Fluorescence readings were normalized to the growth absorbance and fold changes were calculated with respect to the control. The effect of other tryptophan metabolites such as tryptamine, indole-3-acetic acid and indole-3-pyruvic acid was also investigated, on *hilA* expression at a concentration of 1 mM. For investigating synergism between indole and SCFAs, a mixture of SCFAs at published concentrations in cecal luminal contents (110 mM sodium acetate, 70 mM sodium propionate and 20 mM sodium butyrate) was used [21]. Cecal indole concentrations, as reported in [22], of 100 μM and 250 μM were tested. These lower concentrations of indole were used so that significant changes upon SCFA addition can be detected without saturation of the response. To control for osmolarity changes introduced by addition of sodium salts of SCFAs, 200 mM NaCl was used. All experiments were performed in duplicate and repeated with at least three biological replicates.

### Gene expression using RT-qPCR

RNA extraction and RT-qPCR protocol was followed as described in [26]. Briefly, RNA was extracted from late exponential phase cultures (O.D._600nm_ ~ 1.0) using the Qiagen RNeasy Mini kit as per manufacturer’s protocol. DNase treatment of the extracted RNA was carried out using Ambion Turbo DNase kit to remove gDNA contamination. Primers for RT-PCR were designed using the PrimerQuest tool (Integrated DNA Technologies, Inc). PCR was performed followed by agarose gel electrophoresis to determine whether gDNA was effectively eliminated. cDNA was then synthesized using Invitrogen First Strand Synthesis kit as per manufacturer’s instructions. SYBR green master mix from Life Technologies was used to set up qPCR and the run was carried out in Roche LightCycler 96. C_t_ values were obtained using standard procedure and fold change calculated using the ΔΔC_t_ method [54].

### *In-vivo* competitive index experiment

Female C57BL/6 mice (6–8 weeks old) were obtained from The Jackson Laboratories (Bar Harbor, ME). All mice were housed in specific pathogen-free conditions and cared for in accordance with Texas A&M Health Science Center and System Institutional Animal Care and Use Committee guidelines. All *in vivo* experiments in this study were approved by this committee. Wild-type *Salmonella* ATCC14208 (naladixic acid sensitive) and a naladixic acid resistant isogenic strain were cultured to exponential phase in the absence and presence of 1 mM indole, respectively. The Nal^R^ strain has been reported to show similar virulence and *in vivo* persistence to the wild-type ATCC14028 in murine models [51]. The indole treated bacterial inocula had the same viability counts (CFUs) as the control solvent-treated inocula. All cultures were gently washed and resuspended in phosphate buffer saline, and mixed together in equal ratio based on O.D._600_ prior to gastric gavage administration. Five mice were used for each group at each time point and the experiment was repeated for two infection doses. Approximately ~ 5 × 10^7^ (low dose LD) and ~ 5 × 10^8^ cells (high dose HD) were gavaged with feeding needles (22 × 11/2 with 11/4 mm ball, no. 7920, Popper & Sons, Inc., New Hyde Park, NY).

After bacterial challenge, bacterial burden in infected tissues was determined. At different time points (days 1 and 3 post-infection), fecal pellets, liver, spleen, mesenteric lymph nodes, Peyer’s patches and cecum (cecal tissue with luminal contents) were harvested. The samples were homogenized in sterile 0.1% NP40 using a motorized homogenizer (Omni International), the homogenates were serially diluted in sterile 0.1% NP40, and multiple dilutions from each organ were plated in duplicates. Two sets of plates, with and without naladixic acid at a concentration of 50μg/mL, were used to obtain total and Nal^R^ bacterial counts, respectively, in the different tissues. The counts for wild-type bacteria were estimated by subtracting Nal^R^ bacterial counts from total cfu counts that were enumerated by plating. Two types of media (LB or XLD) were used depending on the organ and its inherent microflora. LB agar plates were used for plating samples from the spleen, liver, Peyer’s patches and the mesenteric lymph nodes whereas XLD agar plates were used for fecal and cecum samples to differentiate *Salmonella* (black-colored colonies) from other microbes that are present. Colony forming unit (CFU) counts were determined after overnight incubation at 37°C.

The competitive index (CI) in each sample was calculated as [(cfu of indole-treated strain in the organ/cfu of control strain in the organ)]/[(cfu of indole treated strain used in the inoculum/cfu of control strain used in the inoculum)]. For organs where indole treated *Salmonella* were absent but solvent treated *Salmonella* were present, CI was calculated assuming a cfu of 1 for the indole treated *Salmonella*.

### Statistical analysis

Graph Pad Prism, version 5.0, software was used for statistical analysis and plotting the competitive index data. Wilcoxon Matched Pair signed-rank non-parametric test was used to determine significance of difference between the numbers of two groups: indole-treated and the control (solvent-treated). These observations are paired as they are dependent on the mouse they infected; however, each pair is independent as the observations are obtained from individual (independent) animals. Student’s t-test was performed for the measured values of the *in-vitro* experiments and *p* < 0.05 was considered as statistically significant.

## Supporting information

S1 Fig *In vivo* competition assays in C57BL/6 mice with indole-treated *Salmonella*. The box and whisker representation of data for recovery (cfus/organ) of the indole-treated and non-treated *Salmonella* in different organs harvested from infected mice (n = 5) at days 1 and 3 post inoculation. The box extends from the 25th to the 75th percentile and the whiskers go down to the smallest value and up to the largest. The line in the middle of the box represents the median. Two inoculum doses were tested- low dose (LD ~5 × 10^7^ cfu) and high dose (HD ~5 × 10^8^ cfu) and several organs—cecum, Peyer’s patches, spleen, liver and mesenteric lymph nodes—were harvested. Feces were collected prior to euthanization. The organs were homogenized and dilutions were plated to obtain cfu counts. Organs from LD group mice harvested on day 1 (**S1A**) post inoculation and day 3 (**S1B**) post inoculation. Organs from HD group mice harvested on day 1 (**S1C**) post inoculation and day 3 (**S1D**) post inoculation. * denotes significantly lower (p < 0.05) recovery of indole-treated *Salmonella* relative to solvent-treated *Salmonella*, using the Wilcoxon matched pair test.
(PPTX)

S2 Fig Effect of indole on *Salmonella* swimming motility in WT and Δ*sdiA* strain. Swimming motility assay observations of *Salmonella* (A) WT at 30°C, (B) Δ*sdiA* strain at 30°C and (C) Δ*sdiA* strain at 37°C. Data shown are the measured halo diameters for the different test conditions—no additive, solvent and 1 mM indole at 8 h post-spotting. Diameters were measured using Vernier calipers. Δ*motA* was spotted on swimming motility agar plates as a negative control for motility. Column bars depict mean (n = 4) and error bars represent SD.
(PPTX)

S3 Fig Invasion of epithelial cells and invasion and intracellular survival within macrophages with indole-treated *Salmonella* Δ*sdiA*. Invasion in HeLa epithelial cell line (A) with *Salmonella* treated with or without 1mM indole. Invasion (B) and intracellular survival (C) in J774A.1 cells. A MOI of 100:1 was used for HeLa cells and a MOI of 10:1 was used for J774A.1 macrophages. Data shown are % invasion or survival fold changes, relative to the invasion, normalized to the solvent-treated control. Column bars depict mean (n = 3) and error bars represent SD.
(PPTX)

S4 Fig Complementation of *phoQ* on plasmid. The Δ*phoQ* mutation was generated in the *hilA* reporter and complemented with pCA24N plasmid encoding *phoQ*. The WT, Δ*phoQ* and the Δ*phoQ*+pCA24NStm*phoQ* reporter strains were treated overnight with and without 1 mM indole and the ß-gal activity was measured in exponential phase cultures after dilution. Data shown are the mean fold decrease (n = 3) in expression with indole-treatment relative to the solvent-treated control and error bars represent SD. (*, *p* < 0.05 and **, *p* < 0.005).
(PPTX)

S5 Fig Effect of tryptophan metabolites on *hilA* expression. SPI-1 reporter strain for *hilA* was treated overnight with and without 1 mM tryptophan metabolites: tryptamine, indole-3-acetic acid, indole-3-pyruvic acid and indole, and the ß-gal activity was measured. Data shown are the mean fold decrease (n = 3) in expression of *hilA* with treatment relative to the solvent-treated control which was statistically significant with *p* < 0.05. Error bars represent SD.
(PPTX)

S1 Table SPI-2 gene expression. (PPTX)
