# Supplementary figures and images for: The microbiota metabolite indole inhibits Salmonella virulence: Involvement of the PhoPQ two-component system
Source: PLoS One. 2018 Jan 17;13(1):e0190613. doi: 10.1371/journal.pone.0190613 (PMC5771565; doi:10.1371/journal.pone.0190613)

## Slide 1
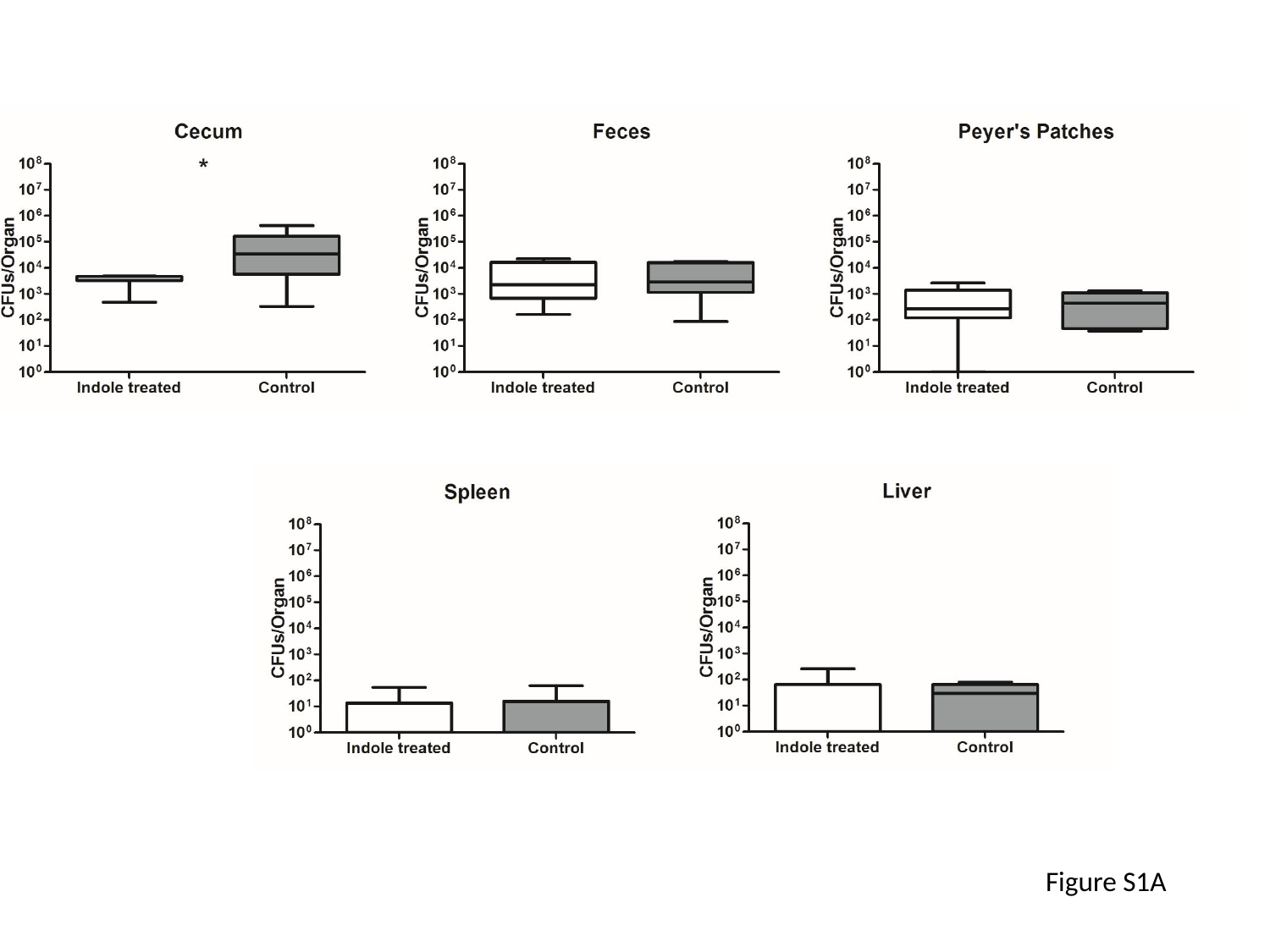

Figure S1A

## Slide 2
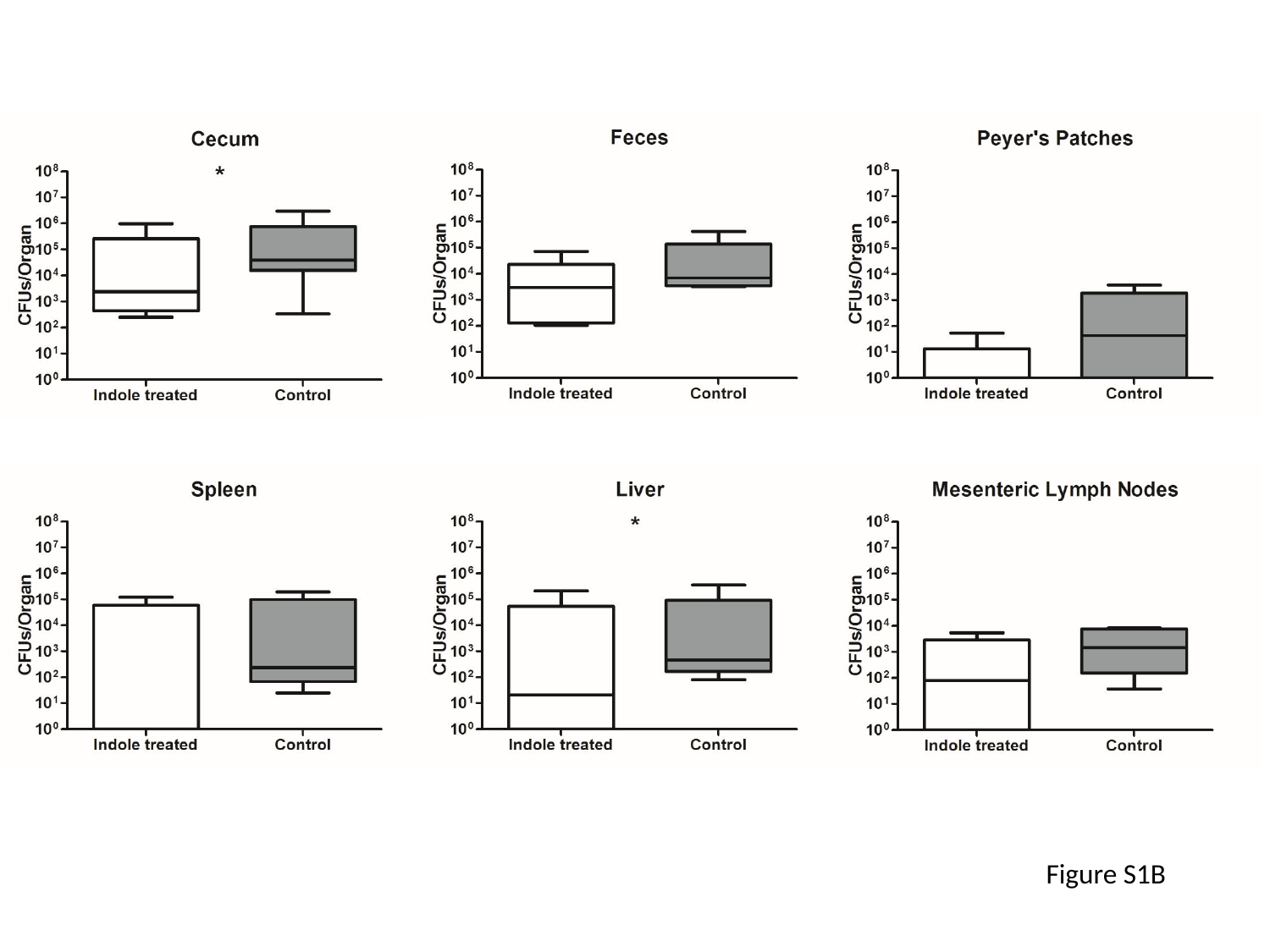

Figure S1B

## Slide 3
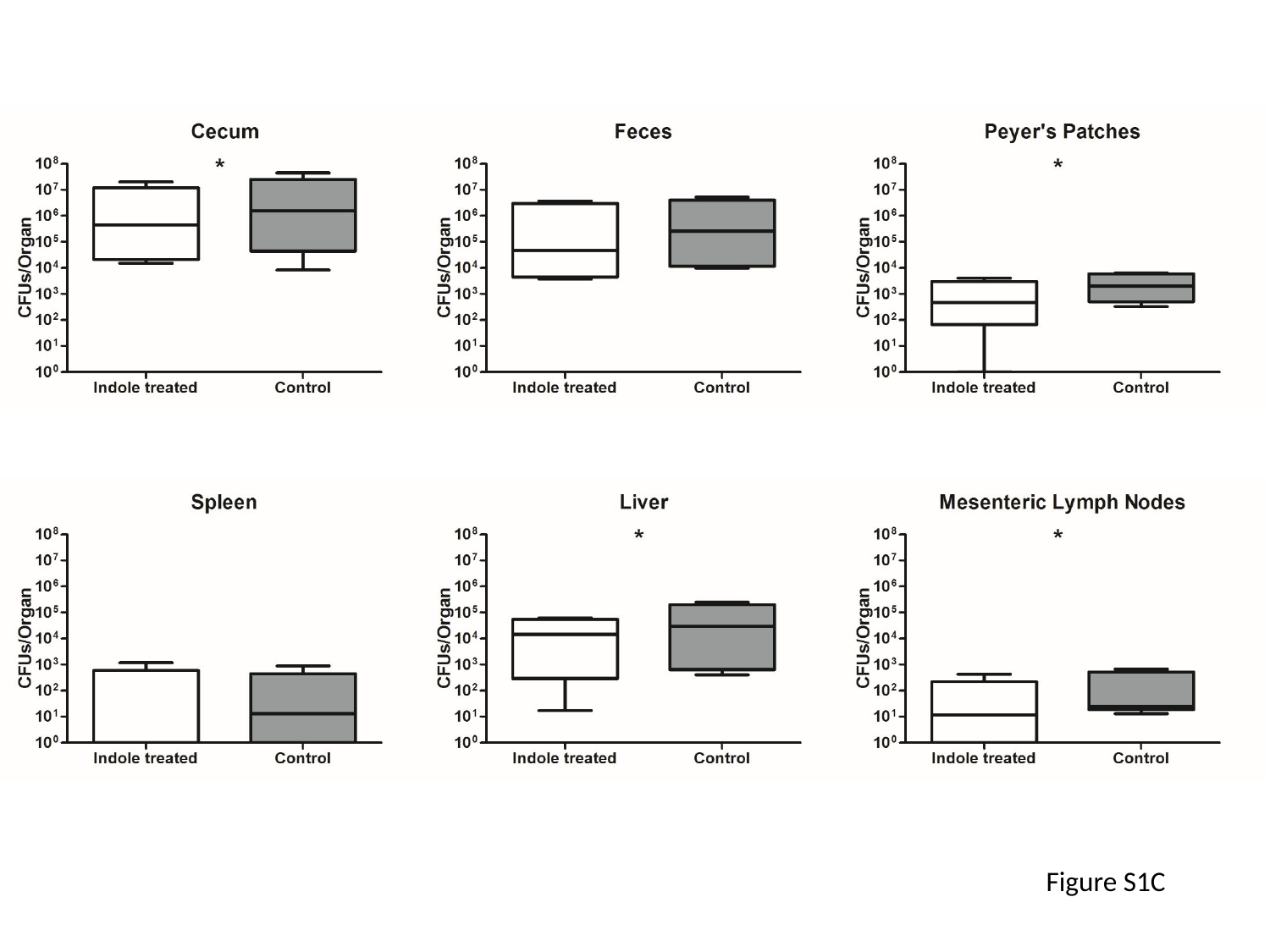

Figure S1C

## Slide 4
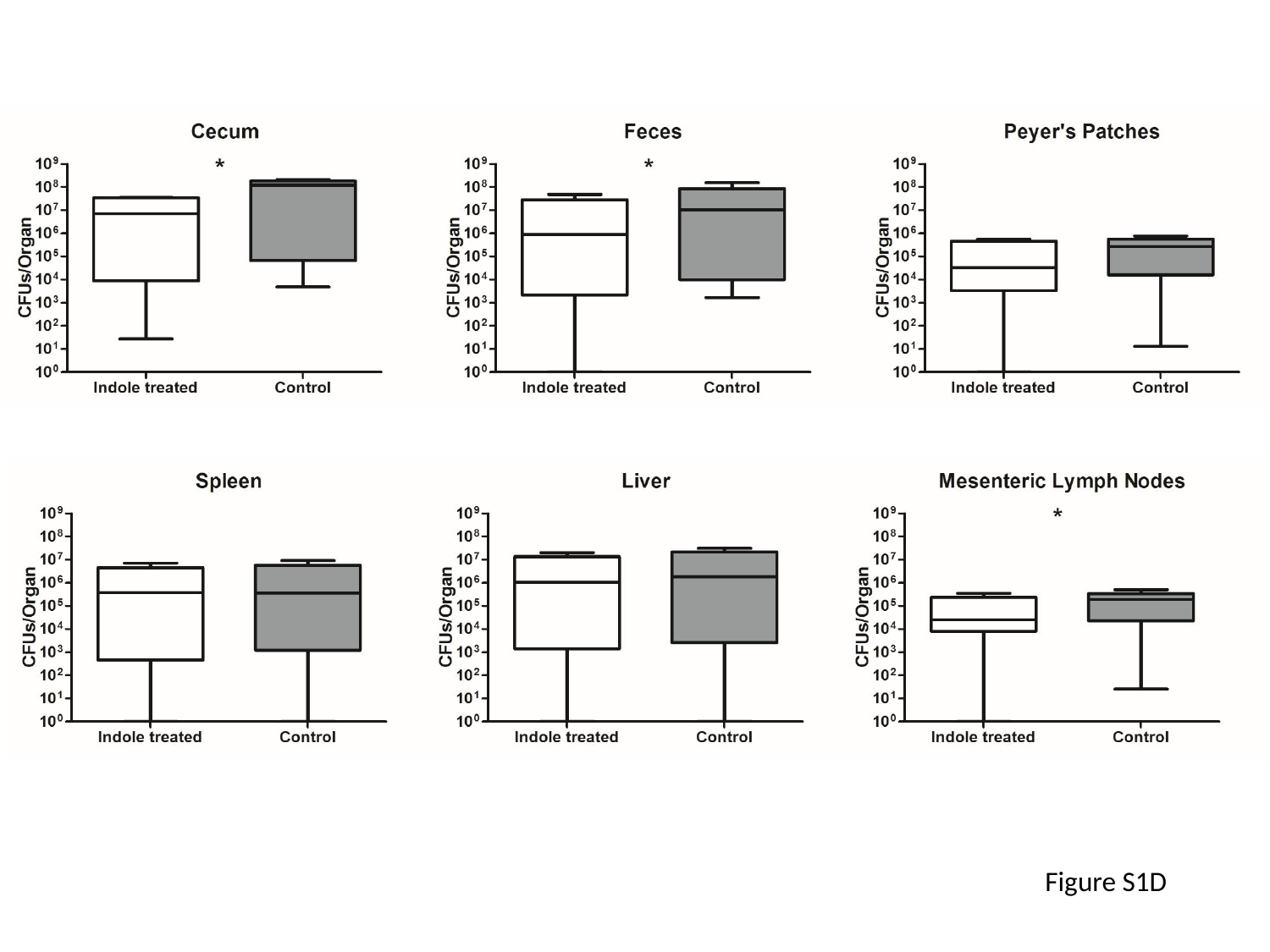

Figure S1D

Supplement: S1 Fig — The box and whisker representation of data for recovery (cfus/organ) of the indole-treated and non-treated Salmonella in different organs harvested from infected mice (n = 5) at days 1 and 3 post inoculation. The box extends from the 25th to the 75th percentile and the whiskers go down to the smallest value and up to the largest. The line in the middle of the box represents the median. Two inoculum doses were tested- low dose (LD ~5 × 10^7^ cfu) and high dose (HD ~5 × 10^8^ cfu) and several organs—cecum, Peyer’s patches, spleen, liver and mesenteric lymph nodes—were harvested. Feces were collected prior to euthanization. The organs were homogenized and dilutions were plated to obtain cfu counts. Organs from LD group mice harvested on day 1 (S1A) post inoculation and day 3 (S1B) post inoculation. Organs from HD group mice harvested on day 1 (S1C) post inoculation and day 3 (S1D) post inoculation. * denotes significantly lower (p < 0.05) recovery of indole-treated Salmonella relative to solvent-treated Salmonella, using the Wilcoxon matched pair test. (PPTX) [file pone.0190613.s001.pptx]

## Slide 1
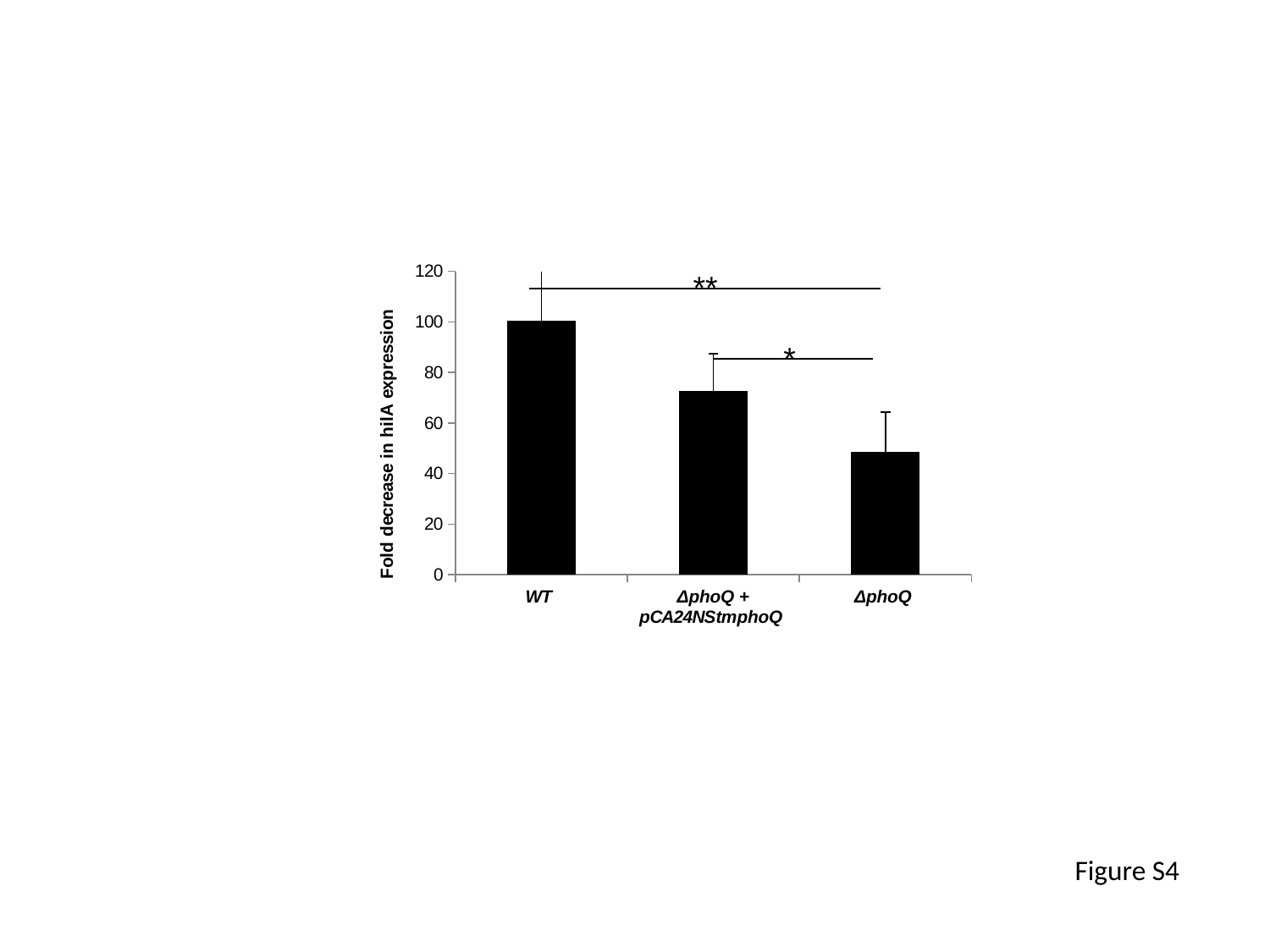

### Chart
| Category | |
|---|---|
| WT | 100.6151291510313 |
| ΔphoQ + pCA24NStmphoQ | 72.81353839617516 |
| ΔphoQ | 48.52086887910154 |**
*
Figure S4

Supplement: S4 Fig — The ΔphoQ mutation was generated in the hilA reporter and complemented with pCA24N plasmid encoding phoQ. The WT, ΔphoQ and the ΔphoQ+pCA24NStmphoQ reporter strains were treated overnight with and without 1 mM indole and the ß-gal activity was measured in exponential phase cultures after dilution. Data shown are the mean fold decrease (n = 3) in expression with indole-treatment relative to the solvent-treated control and error bars represent SD. (*, p < 0.05 and **, p < 0.005). (PPTX) [file pone.0190613.s004.pptx]

## Slide 1
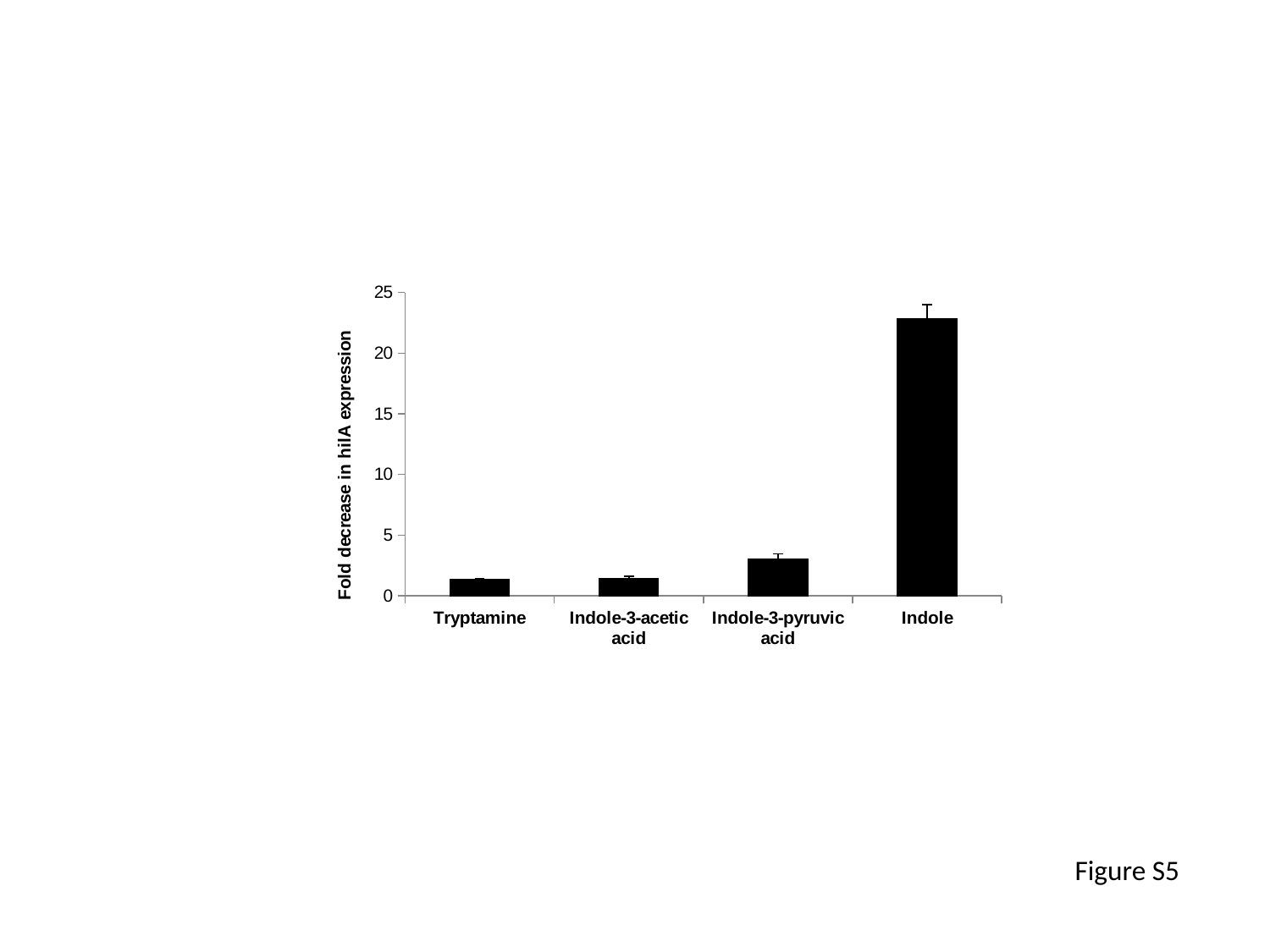

### Chart
| Category | |
|---|---|
| Tryptamine | 1.344754833920823 |
| Indole-3-acetic acid | 1.47378851205285 |
| Indole-3-pyruvic acid | 3.015945555856145 |
| Indole | 22.84789785330464 |Figure S5

Supplement: S5 Fig — SPI-1 reporter strain for hilA was treated overnight with and without 1 mM tryptophan metabolites: tryptamine, indole-3-acetic acid, indole-3-pyruvic acid and indole, and the ß-gal activity was measured. Data shown are the mean fold decrease (n = 3) in expression of hilA with treatment relative to the solvent-treated control which was statistically significant with p < 0.05. Error bars represent SD. (PPTX) [file pone.0190613.s005.pptx]

## Slide 1
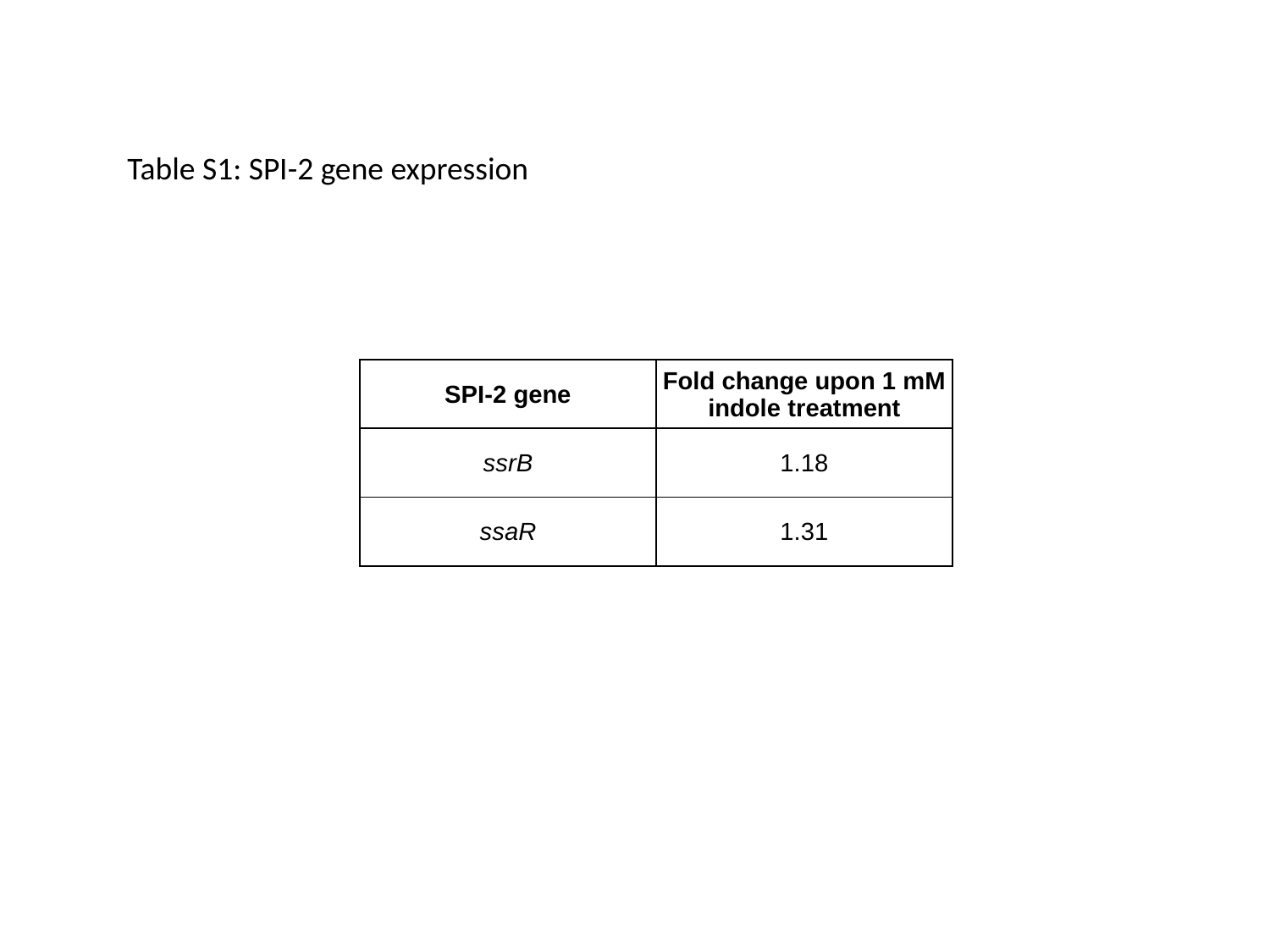

# Table S1: SPI-2 gene expression
| SPI-2 gene | Fold change upon 1 mM indole treatment |
| --- | --- |
| ssrB | 1.18 |
| ssaR | 1.31 |

Supplement: S1 Table — (PPTX) [file pone.0190613.s006.pptx]
