# Supplementary material for: The microbiota metabolite indole inhibits Salmonella virulence: Involvement of the PhoPQ two-component system
Source: PLoS One. 2018 Jan 17;13(1):e0190613. doi: 10.1371/journal.pone.0190613 (PMC5771565; doi:10.1371/journal.pone.0190613)
Supplement: S2 Fig — Swimming motility assay observations of Salmonella (A) WT at 30°C, (B) ΔsdiA strain at 30°C and (C) ΔsdiA strain at 37°C. Data shown are the measured halo diameters for the different test conditions—no additive, solvent and 1 mM indole at 8 h post-spotting. Diameters were measured using Vernier calipers. ΔmotA was spotted on swimming motility agar plates as a negative control for motility. Column bars depict mean (n = 4) and error bars represent SD. (PPTX) [file pone.0190613.s002.pptx]

## Slide 1
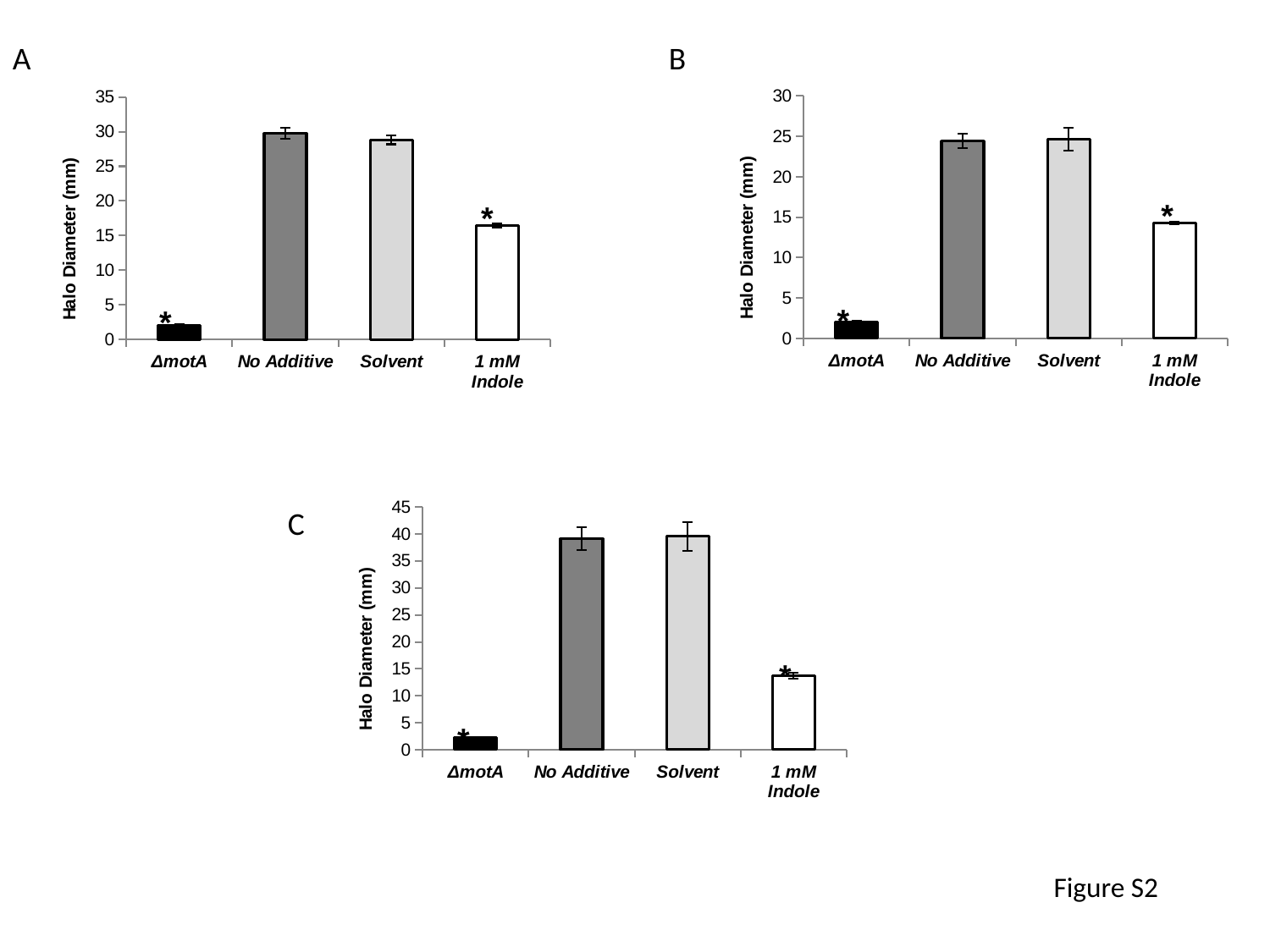

A
B
### Chart
| Category | |
|---|---|
| ΔmotA | 2.05 |
| No Additive | 24.42499999999999 |
| Solvent | 24.625 |
| 1 mM Indole | 14.3 |*
*
### Chart
| Category | |
|---|---|
| ΔmotA | 2.05 |
| No Additive | 29.72499999999999 |
| Solvent | 28.8 |
| 1 mM Indole | 16.45 |*
*
### Chart
| Category | ΔSdiA |
|---|---|
| ΔmotA | 2.2 |
| No Additive | 39.125 |
| Solvent | 39.6 |
| 1 mM Indole | 13.7 |*
*
C
# Figure S2
