# Supplementary material for: The microbiota metabolite indole inhibits Salmonella virulence: Involvement of the PhoPQ two-component system
Source: PLoS One. 2018 Jan 17;13(1):e0190613. doi: 10.1371/journal.pone.0190613 (PMC5771565; doi:10.1371/journal.pone.0190613)
Supplement: S3 Fig — Invasion in HeLa epithelial cell line (A) with Salmonella treated with or without 1mM indole. Invasion (B) and intracellular survival (C) in J774A.1 cells. A MOI of 100:1 was used for HeLa cells and a MOI of 10:1 was used for J774A.1 macrophages. Data shown are % invasion or survival fold changes, relative to the invasion, normalized to the solvent-treated control. Column bars depict mean (n = 3) and error bars represent SD. (PPTX) [file pone.0190613.s003.pptx]

## Slide 1
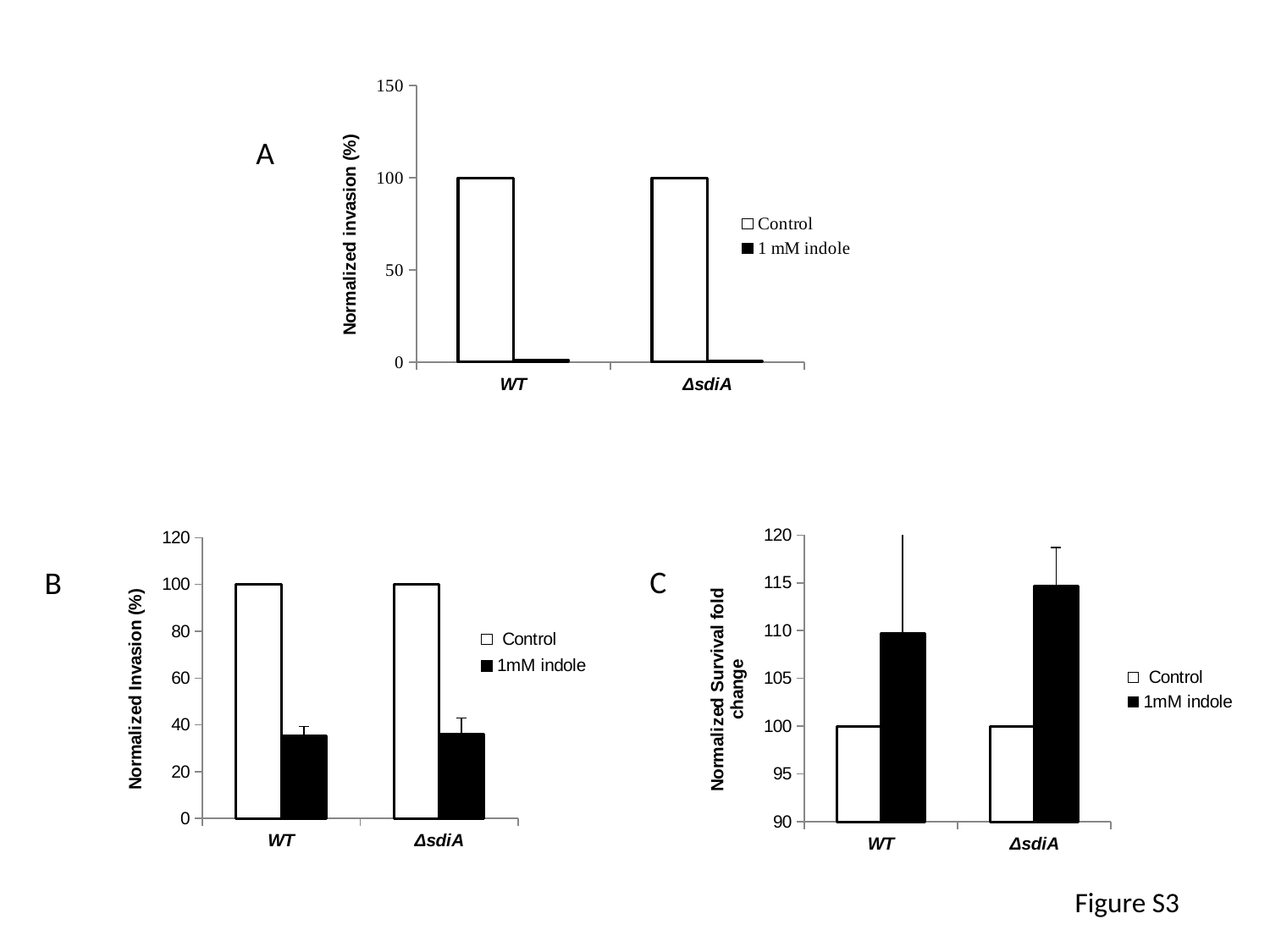

### Chart
| Category | Control | 1 mM indole |
|---|---|---|
| WT | 100.0 | 0.866364236337101 |
| ΔsdiA | 100.0 | 0.541413654154955 |A
### Chart
| Category | Control | 1mM indole |
|---|---|---|
| WT | 100.0 | 35.38260771208645 |
| ΔsdiA | 100.0 | 36.05035358891426 |
### Chart
| Category | Control | 1mM indole |
|---|---|---|
| WT | 100.0 | 109.7437623022418 |
| ΔsdiA | 100.0 | 114.697853489278 |C
B
Figure S3
